# Epidermal Differentiation Genes of the Common Wall Lizard Encode Proteins with Extremely Biased Amino Acid Contents

**DOI:** 10.3390/genes15091136

**Published:** 2024-08-28

**Authors:** Karin Brigit Holthaus, Attila Placido Sachslehner, Julia Steinbinder, Leopold Eckhart

**Affiliations:** Department of Dermatology, Medical University of Vienna, 1090 Vienna, Austria

**Keywords:** gene family, gene duplication, reptiles, lizards, keratinocytes, disulfide bonds, evolution, epidermis, skin

## Abstract

The epidermal differentiation complex (EDC) is a cluster of genes that code for protein components of cornified cells on the skin surface of amniotes. Squamates are the most species-rich clade of reptiles with skin adaptations to many different environments. As the genetic regulation of the skin epidermis and its evolution has been characterized for only a few species so far, we aimed to determine the organization of the EDC in a model species of squamates, the common wall lizard (*Podarcis muralis*). By comparative genomics, we identified EDC genes of the wall lizard and compared them with homologs in other amniotes. We found that the EDC of the wall lizard has undergone a major rearrangement leading to a unique order of three ancestral EDC segments. Several subfamilies of EDC genes, such as those encoding epidermal differentiation proteins containing PCCC motifs (EDPCCC) and loricrins, have expanded by gene duplications. Most of the EDPCCC proteins have cysteine contents higher than 50%, whereas glycine constitutes more than 50% of the amino acid residues of loricrin 1. The extremely biased amino acid compositions indicate unique structural properties of these EDC proteins. This study demonstrates that cornification proteins of the common wall lizard differ from homologous proteins of other reptiles, illustrating the evolutionary dynamics of diversifying evolution in squamates.

## 1. Introduction

The epidermis of the skin has evolved as a protective tissue that is critical for the survival of land-dwelling vertebrates. In particular, the epidermis of reptiles is adapted to low environmental humidity and exposure to mechanical stress. The surface of the epidermis, which is in direct contact with the environment, consists of dead epithelial cells. These cells are almost entirely filled with cytoskeletal intermediate filaments and heavily cross-linked proteins that endow the skin surface with resistance to mechanical stress and contribute to the protection against water loss [1]. The formation and renewal of this superficial cell layer is achieved by the differentiation of epidermal keratinocytes and particularly by its last step, that is, cornification [2,3]. In line with the substantial variability in integumentary phenotypes of fully terrestrial vertebrates [4,5,6], the central role of the proteinaceous core of the skin barrier and its requirement for cross-linking mechanisms are conserved, whereas major differences exist with regard to the sequences and number of cornification-associated proteins in the diverse clades of reptiles, birds and mammals [7,8].

The epidermal differentiation complex (EDC) is a cluster of genes that are expressed during terminal differentiation and encode structural protein components of keratinocytes [7,8]. The EDC was first characterized in humans and later described in other mammals, and also in reptiles and birds [9,10,11,12,13,14,15,16,17,18]. In all amniotes investigated so far, the EDC is flanked by S100A genes, which contain an S100 domain. The same domain is also present at the amino-terminus of S100 fused-type proteins (SFTPs), such as filaggrin, trichohyalin and scaffoldin [19]. SFTPs are characterized by a large carboxy-terminal segment of repetitive amino acid sequences, which differ substantially between individual SFTPs. Mutations of human filaggrin are associated with ichthyosis vulgaris and atopic dermatitis [20,21]. The second main class of EDC genes besides SFTP genes is called single-coding-exon EDC genes, short SEDCs, to indicate a common feature of their gene organization that distinguishes them from S100A and SFTP genes, which contain protein coding sequences in two exons. All the aforementioned EDC genes contain a non-coding exon at the 5’-end that is preceded by a promoter with a canonical TATA box and binding sites for transcription factors such as AP-1 [19]. SEDC proteins display a remarkable diversification of sequences. Corneous beta proteins (CBPs) are characterized by beta sheet-forming segments that are predicted to facilitate the dimerization of CBPs and the formation of filaments [22,23]. The other SEDCs are not predicted to fold into canonical domains because they have amino acid sequences of low complexity and, in many cases, display an enrichment of one or few amino acid residues [24]. Examples for the latter are loricrin (glycine-rich), small proline-rich proteins (SPRRs) and epidermal differentiation cysteine-rich protein (EDCRP) [13]. Many EDC proteins undergo covalent cross-linking via transglutamination, which connects glutamine (Q) and lysine (K) residues [1,25], and disulfide bond formation between cysteine residues [1,26]. Accordingly, besides keratin intermediate filaments, EDC proteins are predicted to function as molecular building blocks of the proteinaceous material at the skin surface.

Squamates are by far the most diverse clade of reptiles with more than 11,000 species [27,28]. They utilize the most complex mode of epidermal regeneration, which depends on tightly controlled epithelial cell proliferation and differentiation over time, and the coordinated shedding of the outer layers of the epidermis [6,29,30,31]. A series of studies in the past twenty years have revealed many molecular constituents and regulators of epidermal differentiation in squamates [12,13,15,17,32]. Major lines of research have been dedicated to keratin intermediate filaments [32,33], corneous beta-proteins, also known as beta-keratins [12,23,34], and proteins encoded by genes that are clustered in the so-called epidermal differentiation complex (EDC) [13,15,17].

The aim of the present study was to characterize the EDC of the common wall lizard (*Podarcis muralis*), a species of the family *Lacertidae* that naturally inhabits Southern Europe and has been introduced to other parts of Europe and North America. It colonizes various habitats and has been used as a model for evolutionary diversification. Several studies have addressed the function and evolution of the skin color polymorphisms of the common wall lizard [35,36,37]. In other studies, *P. muralis* served as a model for tissue regeneration and skin biology [38,39,40,41,42]. Recently, the interactions of epidermal keratins of this species have been studied [33]. A limited amount of information is available on EDC proteins of *P. muralis* [17,40]. Here, we determined the organization of the complete EDC of the common wall lizard.

## 2. Materials and Methods

### 2.1. Identification of EDC Genes in Genome Sequences

The genome sequence of the common wall lizard (*P. muralis*) [35] was analyzed for EDC genes according to a published gene search and gene prediction approach [13,14]. Database accession numbers of EDC genes of the wall lizard are listed in Table S2. In brief, tBLASTn [43] searches were performed with EDC proteins of other squamates as queries [13,15]. Importantly, the filter for low-complexity regions was deactivated because low sequence complexity is a typical feature of EDC proteins [13]. Additional genes were identified by in silico translation of the genomic region homologous to the EDC in other reptiles. De novo-predicted EDC genes were used as queries in searches for similar proteins in the same and in other species. The organization of the EDC was compared between *P. muralis* and the green anole lizard (*Anolis carolinensis*) [44]. Sequences were aligned with MultAlin [45] and MUSCLE [46], followed by manual adjustments. ProtParam was used to determine the percentages of the amino acid residues in the proteins [47].

For the validation of expression of a newly predicted gene, the amino acid sequence of the corresponding protein was used as a query in a tBLASTn search in the transcriptome, as determined by RNA-seq of *P. muralis* adult male skin (NCBI GenBank sequence read archive accession: SRX5274937) [35].

### 2.2. Proteomics

Tissue samples from green anole lizards (*A. carolinensis*) were available from a previous study of our research group, having been kept at −80 °C [13]. Toes were isolated and lysed in a buffer containing 30 mM Tris, 7 M urea (VWR, Radnor, PA, USA), 2 M thiourea (Sigma-Aldrich, St. Louis, MI, USA), 4% CHAPSO (Pierce, Waltham, MA, USA) and 0.2 M dithiothreitol (DTT) at 70 °C for three hours. The samples were processed in a Precellys homogenizer (VWR) and subsequently stored at −80 °C. For proteomic analysis, the samples were further reduced with 0.5 M DTT, alkylated with iodoacetamide (1 M, Sigma) and bound to SP3 beads (GE Healthcare, Chicago, IL, USA) at a 10:1 ratio of beads:protein. After washing with 80% ethanol and acetonitrile, the samples were digested with trypsin/LysC (Promega, Fitchburg, WI, USA) at a 1:25 enzyme:protein ratio in 50 mM ammoniumbicarbonate (Sigma) overnight at 37 °C. After elution peptides were desalted using Pierce Peptide Desalting columns (ThermoFisher, Waltham, MA, USA), dried in a vacuum concentrator and reconstituted in 0.1% TFA. The peptide concentration was measured using the Pierce Quantitative Colorimetric Peptide Assay (Thermo Fisher). The samples were analyzed on an Ultimate 3000 RSLC nano coupled directly to an Exploris 480 with FAIMSpro (all Thermo Fisher) according to a previously published protocol with modifications [48].

The samples were injected onto a reversed-phase C18 column (50 cm × 75 µm i.d., packed in-house) and eluted with a gradient of 4% to 38% mobile phase B over 94 min by applying a flow rate of 230 nL/min. MS scans were performed in the range of *m*/*z* 375–1650 at a resolution of 60,000 (at *m*/*z* = 200). MS/MS scans were performed, choosing a resolution of 15,000; normalized collision energy of 29%; isolation width of 1.4 *m*/*z* and dynamic exclusion of 90 s. Two different FAIMS voltages were applied (−40 V and −60 V) with a cycle time of 1.5 s per voltage. FAIMS was operated in standard resolution mode with a static carrier gas flow of 4.1 L/min.

The acquired raw MS data files were processed and analyzed using ProteomeDiscoverer (v2.4.0.305, Thermo Fisher). SequestHT was used as the search engine and the following parameters were chosen: database—*A. carolinensis* (taxonomy ID: 28377, reference proteome: UP000001646, downloaded from Uniprot on 1 July 2024); enzyme—trypsin; maximum missed cleavage sites—2; static modifications—carbamidomethyl (C); dynamic modifications—oxidation (M), acetyl (protein N-terminus), Met-loss (M) and Met-loss+Acetyl (M); precursor mass tolerance—10 ppm; fragment mass tolerance—0.02 Da. Only peptides and proteins with FDR < 0.01 are reported, and single peptide IDs were excluded from the dataset.

## 3. Results

### 3.1. The Organization of the EDC of the Common Wall Lizard Differs from the Canonical EDC Structure

Only few predictions for genes of the EDC of *P. muralis* were available in GenBank. To obtain a complete or close-to-complete overview of the EDC, we performed tBLASTn searches using EDC proteins of *A. carolinensis* as queries and predicted genes de novo on the basis of translations of the EDC nucleotide sequence according to a published approach [13]. Preliminary names were assigned to the newly identified genes of the EDC, using a previously suggested naming system in which the letters ED (for Epidermal Differentiation) are followed by a term indicating either a specific amino acid composition or a peculiar sequence motif of the protein encoded by the gene. For better readability, only abbreviated names are used in the text and figures. Full names are listed in Table S1. Accession numbers and genomic positions of EDC genes are listed in Table S2 and amino acid sequences are provided in Figure S1.

**Figure 1:**
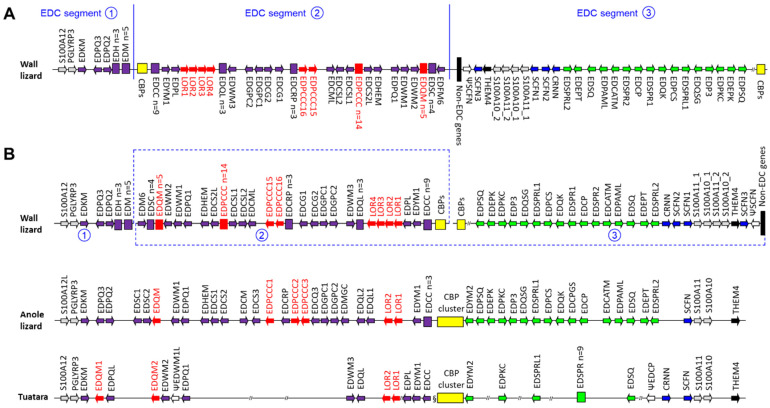
The epidermal differentiation complex (EDC) of the common wall lizard has an untypical organization, suggesting a rearrangement of EDC genes during evolution. (**A**) The EDC of the wall lizard (*P. muralis*) is schematically depicted, whereby the genes are shown as arrows pointing in the direction of transcription. Arrays of 3 or more genes of the same family are shown as boxes. Genes discussed in the text are highlighted red. Other colors important indicate gene families (yellow, CBPs, corneous beta-proteins; blue, SFTPs, 100 fused-type proteins; purple and green, subgroups of SEDCs, single-coding-exon EDC genes). Note that three segments (indicated by encircled numbers 1–3, separated by blue vertical lines) of the EDC of the wall lizard have undergone a rearrangement. Non-EDC genes are shown in black. (**B**) The genes of the EDC of the wall lizard were aligned with the EDC of the anole lizard (*A. carolinensis*) and the tuatara (*Sphenodon punctatus*). Members of gene families have been numbered in each species without inferring 1:1 orthology to genes of the same number in other species. The symbol // is used to indicate gaps in the scaffold assembly, whereas § indicates the discontinuity of the assembly with genes on different scaffolds. The schematic depiction is not drawn to scale. CBP, corneous beta-protein. Gene names are explained in Table S1.

The comparison of the relative positions of EDC genes in *P. muralis* and other lepidosaurs indicates that the EDC of the wall lizard has undergone a major rearrangement. Three segments (marked by the numbers 1, 2 and 3) are present in an untypical order and orientation to each other in the wall lizard (Figure 1A). However, the arrangement within the three segments is largely the same (conserved synteny) as in other lepidosaurs [13,15,17] (Figure 1B). Many but not all of the EDC proteins are conserved in the wall lizard and the anole lizard. Remarkably, we found that the wall lizard contains four SFTP genes (cornulin and three scaffoldins/trichohyalin-likes), which is one more than the maximum number of SFTPs previously identified in squamates [15].

### 3.2. The EDPCCCs of the Common Wall Lizard Have Uniquely High Cysteine Contents

**Figure 2:**
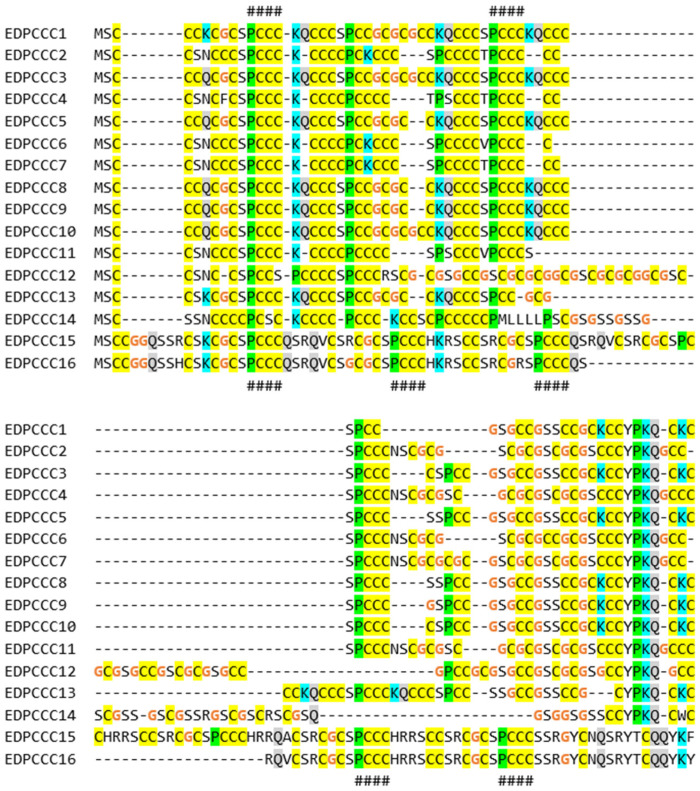
The EDPCCC paralogs of the common wall lizard have a high abundance of cysteine residues over the entire length of the protein. Amino acid sequence alignment of EDPCCC proteins of the common wall lizard (*P. muralis*). Characteristic PCCC motifs are marked with # symbols above and below the sequences. The following amino acid residues are colored as follows: cysteine (C) yellow, lysine (K) cyan, glutamine (Q) grey, proline (P) green shading, and glycine (G) orange font.

We focused our further investigations on subfamilies of EDC genes that (i) have expanded in numbers in the wall lizard as compared to the green anole lizard and (ii) display peculiar features in their amino acid sequences. A strong increase in gene copy number was detected for the family of *EDPCCCs*, which encode proteins containing proline–cysteine–cysteine–cysteine motifs (Figure 2). Three complete and one partial coding sequence of *EDPCCC* paralogs were previously reported for *A. carolinensis* [15]. By mass spectrometry-based proteomic analysis, we could confirm the expression of two EDPCCC paralogs in the toes of *A. carolinensis* (Figure S2A,B). The EDC of the common wall lizard contains 16 *EDPCCC* genes (Figure 2). Remarkably, EDPCCC proteins have a cysteine content above 50%, which is higher than that of any other EDC protein reported so far. Besides the large number of potential sites for disulfide bond formation, Q and K residues as potential sites of transglutamination were also conserved in EDPCCCs (Figure 2).

### 3.3. The EDC of the Wall Lizard Comprises Genes That Encode Loricrin and Corneous Beta-Proteins with Extremely High Glycine Contents

**Figure 3:**
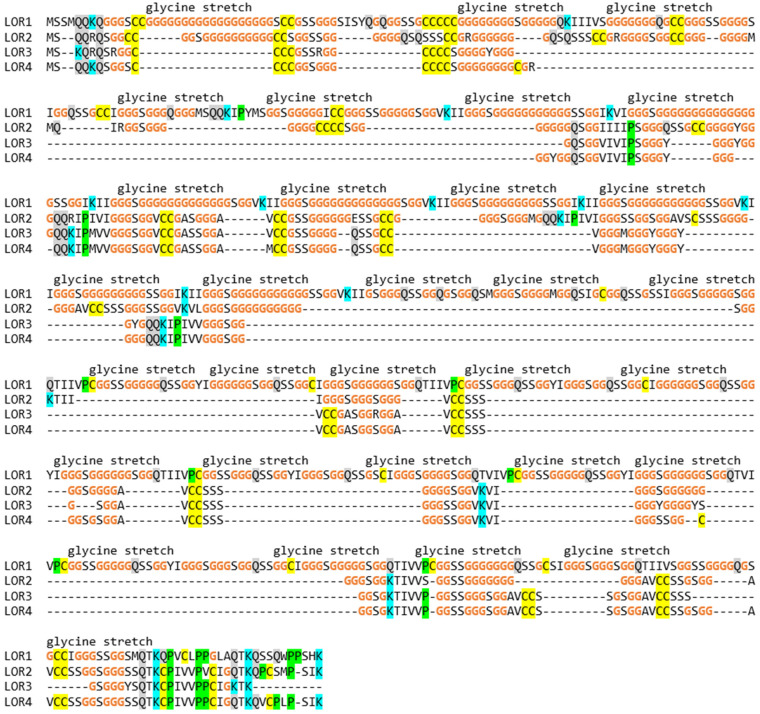
Loricrin proteins of the common wall lizard contain multiple stretches of glycine residues over the entire length of the protein. Amino acid sequence alignment of loricrin proteins of the common wall lizard (*P. muralis*). Amino acid residues C, G, K, P and Q are highlighted by colors as in the previous figure. Glycine-rich stretches of residues are indicated above the sequences. Note that loricrins 2, 3 and 4 are more similar to each other than to loricrin 1.

Loricrin is conserved in amniotes and represents one of the main components of the cornified envelope of terminally differentiated keratinocytes in the human epidermis [13]. Two *loricrin* (*LOR*) genes are present in the EDC of the green anole lizard [13]. Loricrin 1 was immunolocalized in the maturing α-layer of the epidermis of *A. carolinensis* [49], and peptides corresponding to loricrin 2 were detected by proteomic analysis of toes of *A. carolinensis* (Figure S2C). We detected four loricrin genes in the EDC of the common wall lizard. The characteristic organization of loricirin is defined by stretches of glycine and serine residues that are interrupted by 4–7 amino acid residues including isoleucine (I) and valine (V). A similar organization of mammalian loricrins has been proposed to cause the formation of glycine-rich loops, which are highly flexible without assuming a regular secondary structure [50,51]. All four loricrin isoforms contain a carboxy-terminal sequence similar to a motif found in several other SEDC proteins, as reported previously [13] (Figure 3). The glycine contents of the loricrin paralogs of *P. muralis* were higher than in other loricrins reported so far, with glycine accounting for 418 out of 739 amino acid residues of loricrin 1 (glycine content 56.6%).

**Figure 4:**
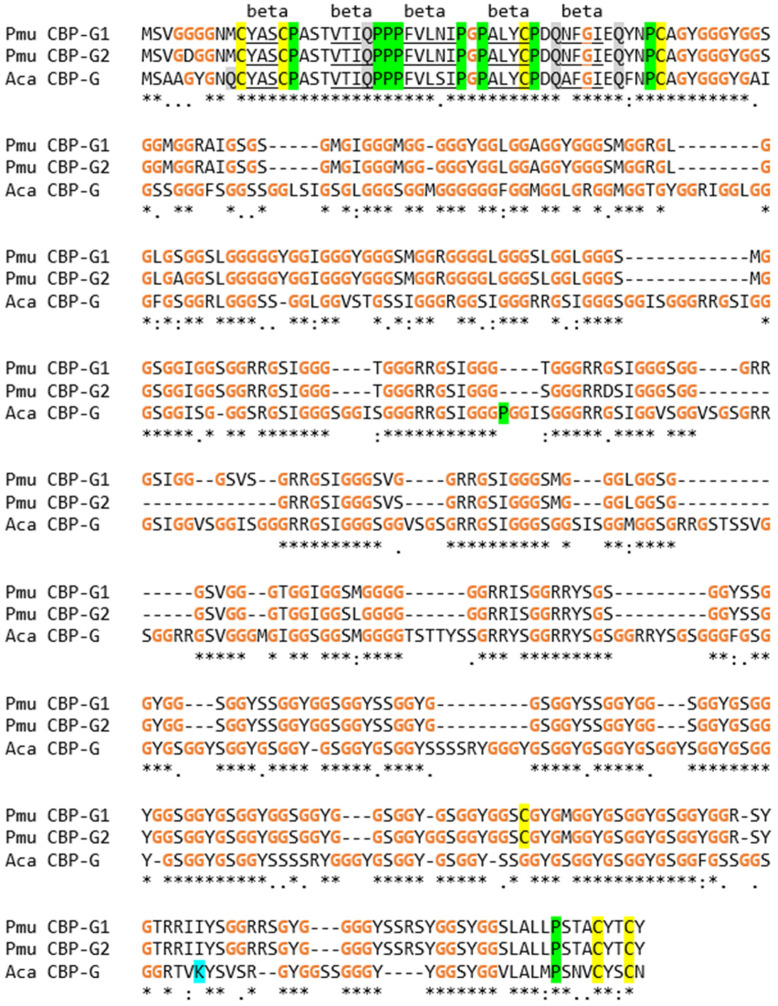
Corneous beta-proteins rich in glycine (CBP-G) proteins encoded by two paralogous genes in the EDC of the common wall lizard. Amino acid sequence alignment of CBP-G proteins of the common wall lizard (*P. muralis*, Pmu) and the green anole lizard (*A. carolinensis*, Aca). Amino acid residues C, G, K, P and Q are highlighted by colors as in the previous figures. Note that Aca CBP-G is encoded by a gene reported as Li-Ac-37 [12]. Dashes were introduced to optimize the alignment. Identical, highly similar and weakly similar residues are marked by “*”, “:” and “.” below the sequences. Amino acid residues predicted to form strands of a beta-sheet are underlined and marked as “beta” above the sequences.

Besides loricrins, two CBPs of the common wall lizard have very high glycine contents. We tentatively named these proteins CBP-G (CBP glycine-rich) 1 and 2. They are orthologs of a single CBP in *A. carolinensis*, which was originally reported as Li-Ac-37 [12]. Amino acid sequence alignment showed a high degree of sequence conservation over the entire length of the proteins (Figure 4). The glycine contents were 49.9% and 49.5% for *P. muralis* CBP-G1 and CBP-G2, respectively. Both values are higher than the glycine content of *A. carolinensis* CBP-G (Li-Ac-37), which is 46.1%.

### 3.4. Gene Duplications Have Led to High Numbers of EDC Protein Paralogs in the Common Wall Lizard

**Figure 5:**
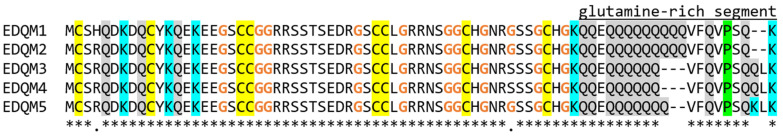
Five EDQM (epidermal differentiation proteins containing a glutamine motif) paralogs of the common wall lizard display a high degree of sequence conservation. Amino acid sequence alignment of EDQMs of the common wall lizard (*P. muralis*). Dashes were introduced to optimize the alignment. Identical and similar residues are marked by “*” and “.” below the sequences. The sequences of EDQM3 and EDQM4 are completely identical. The glutamine (Q)-rich segment at the carboxy-terminus of the proteins is indicated by a line above the sequences. Amino acid residues C, G, K, P and Q are highlighted by colors, as in the previous figures.

Further genes, such as *EDCC*, *EDCRP* and *EDQL*, are present in higher copy numbers in the EDC of the wall lizard than in the EDC of the green anole lizard. Genes tentatively named *EDH1* through *EDH3* and *EDM1* through *EDM6* exist in the wall lizard, but not in the anole lizard (Figure 5). The expression and gene structure of *EDM1* was confirmed by the identification of intron-spanning RNA-sequencing reads from *P. muralis* skin (Figure S3). Gene duplications have given rise to five *EDQM* and nine *EDCC* genes (Table S2, Figure S1). The amino acid sequence alignment of EDQM proteins confirms their high degree of sequence conservation and the presence of 11–13 glutamines within the last 20 residues at the carboxy-terminus of the protein (Figure 5).

**Figure 6:**
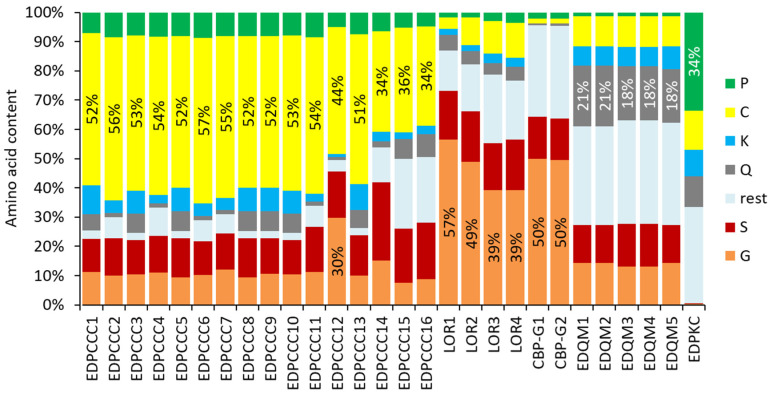
Amino acid compositions of selected SEDC proteins of the common wall lizard (*P. muralis*). The abundance of amino acid residues (% of total residues) is depicted. Colors are explained in the legend on the right.

The comparison of the amino acid contents of a selected subset of EDC proteins demonstrated that several proteins have similar compositions, while enormous differences exist between proteins of different subfamilies (Figure 6). As shown in detail above (Figure 2, Figure 3, Figure 4 and Figure 5), the single amino acids cysteine and glycine account for more than half of the total residues of different EDC proteins. It is noteworthy that potential target sites of transglutamination, namely glutamine and lysine residues, are conserved in most SEDC proteins of such extremely biased amino acid composition (Figure 2, Figure 3 and Figure 5).

## 4. Discussion

This study provides important insights into the epidermal differentiation of a model species of squamate biology, and reveals peculiar features of cornification proteins that are of interest for the diversifying evolution of proteins in general. The advances achieved by this study were made possible by the availability of the genome sequence of the common wall lizard. However, it is important to note that the genes of the EDC are notoriously difficult to predict using the algorithm used at GenBank, as we have shown in previous studies [13,14,15,16,17]. Only the careful search for EDC candidate genes by an established tBLASTn screening and de novo gene prediction approach has allowed us to identify multiple genes that are absent from the current genome annotation of *P. muralis*.

The present analysis of the SEDC and SFTP genes of the wall lizard goes clearly beyond the studies of individual EDC proteins that have been published previously [17,40]. In a study of EDC proteins of the tuatara in comparison to EDC proteins of squamates, we predicted and analyzed the sequences of proline-rich SEDC proteins and the organization of three SFTP genes [17]. This previous report included neither the comprehensive description of all SEDCs and SFTPs of *P. muralis* nor the overall organization of the EDC in this species. With regard to individual EDC proteins, our study was focused on EDC proteins with high cysteine, high glycine or high glutamine contents (Figure 2, Figure 3, Figure 4, Figure 5 and Figure 6). Only a small subset of CBPs were included in this analysis, because few members of this important protein family of *P. muralis* have extreme amino acid contents, thus resembling CBPs in other lepidosaurs [12,52].

The present study has several limitations. Although we have successfully applied the approach of predicting EDC genes in other species [13,14,15,17], the precise structure of the genes remains to be validated. Similarly, the expression pattern of the EDC genes in the skin of different body sites and in other tissues of the common wall lizard remains to be determined. Importantly, changes of expression are expected during the epidermal growth and shedding cycle. The most challenging open research question pertains to the function of individual EDC proteins, which should be investigated either by the deletion of the corresponding genes in vivo or by studying protein interactions in vitro.

The arrangement of EDC genes in the common wall lizards is different from the canonical EDC organization in a wide variety of sauropsids [13,14,15,16,17], and, therefore, must be a lineage-specific feature. Preliminary investigations of other species of *Lacertidae*, namely, *Lacerta agilis* (EDC located on a chromosome with GenBank accession number NC_046328.1) and *Podarcis raffonei* (EDC located on a chromosome with GenBank accession number NC_070617.1), indicate that their EDCs have a similar organization, suggesting a re-arrangement of EDC genes prior to the divergence of *P. muralis* from related species. However, further studies are required to precisely map the rearrangement(s) on a species tree. Importantly, the organization of the EDC of *P. muralis* indicates that the separation of several EDC genes is compatible with the correct regulation of gene expression. Previously, topologically associating domains (TADs) were defined in mammalian EDCs [53,54]. TADs are implicated in the regulation of clustered genes because DNA sequences, such as enhancers and promoters, interact with each other more efficiently within a TAD. It will be interesting to investigate whether the positions of EDC discontinuities of *P. muralis* maintain TADs of the reptilian EDC.

The amino acid compositions of several SEDC proteins of the common wall lizard are extremely biased. Compositional biases are observed in many EDC proteins [7,24], but proteins of the wall lizard are special in comparison to known proteins. Human loricrin has a glycine content of 46.5%, and loricrin 1 of the chicken has a glycine content of 50.8% [13], whereas loricrin 1 of *P. muralis* contains more than 56% glycine. Similarly the cysteine content of EDPCCCs is uniquely high in *P. muralis* and higher than that of ultra-high sulfur proteins of the keratin-associated protein (KRTAP) family [55] and metallothioneins [56]. With more than 55% cysteine residues, EDPCCC2 and EDPCCC6 may be among the most cysteine-rich proteins in general. It is presently unknown whether the enrichments of single amino acids have evolved by chance or under constraints specific for wall lizards. A high cysteine content is predicted to support crosslinking via disulfide bonds, thereby increasing the rigidity of cornified keratinocytes, and an extremely high glycine content leads to intrinsic disorder, which is associated with structural flexibility and interactions with other proteins [24,57].

**Figure 7:**
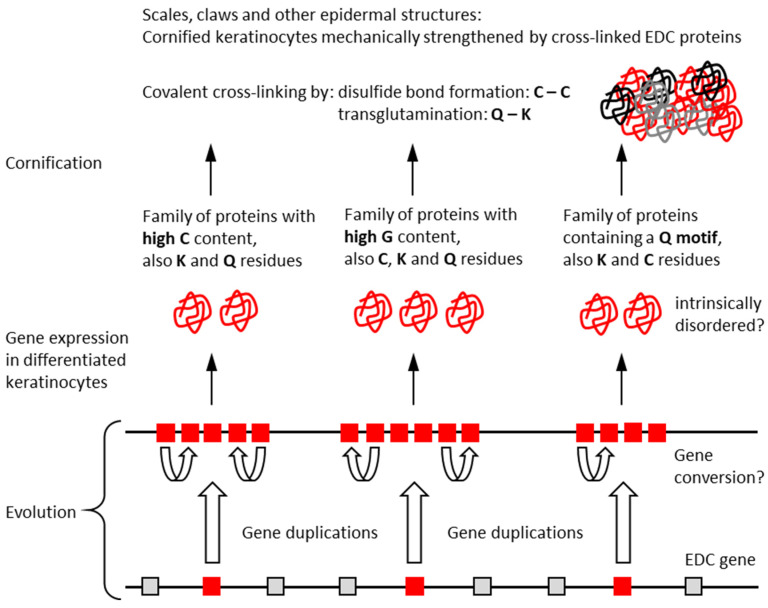
Schematic depiction of the evolution and function of epidermal differentiation complex (EDC) proteins in the common wall lizard (*P. muralis*). EDC protein families discussed in this manuscript are highlighted. Additional proteins (indicated by grey and black lines in the graphic on the top right) are involved in cornification.

The peculiar features of EDC proteins of the common wall lizards indicate important aspects of their evolution and function, which are summarized in Figure 7. First, gene duplications have played a major role in generating multiple copies of genes within EDC subfamilies. Gene conversion may have contributed to the maintenance of sequence similarities within each subfamily, without impairing divergent evolution in different subfamilies. Second, the expression of multiple genes of each family leads to elevated amounts of functionally equivalent proteins. Third, the epidermal differentiation proteins are characterized by biased amino acid compositions and low sequence complexity. These features allow for interactions that essentially depend on single sites, such as disulfide bonds, and largely prevent the folding into conventional protein domains. This suggests that SEDC proteins and the carboxy-terminal segment of SFTPs are intrinsically disordered. The simple beta-sheet of CBPs probably represents the most complex stable structure of SEDC proteins. Fourth, EDC proteins undergo covalent cross-linking by transglutamination and disulfide bond formation to form a largely amorphous proteinaceous matrix, which, together with keratin intermediate filaments, provides mechanical stability to hard epidermal structures such as scales and claws (Figure 7).

The EDC of the common wall lizard was investigated to obtain insights into cornification proteins of an exemplary lacertid species. However, the processes summarized in Figure 7 are not specific for the wall lizard. Most conclusions are rather valid for other squamates, too, or even for sauropsids in general. Our comparison between the common wall lizard and the green anole lizard shows that there are many commonalities in features of the EDC, but also several notable differences. Further detailed analyses of EDC genes in other species of squamates are required to characterize the molecular diversity of the skin in this large taxon.

## Data Availability

Genome sequence data were obtained from NCBI GenBank with accession numbers provided in Table S2. Proteome data were deposited in the PRIDE database under the accession number PXD054063.

## Supplementary Materials

The following supporting information can be downloaded at: https://www.mdpi.com/article/10.3390/genes15091136/s1, Table S1: Abbreviations and full names of EDC genes; Table S2: EDC genes of the common wall lizard; Figure S1: Amino acid sequences of proteins encoded by EDC genes of the common wall lizard; Figure S2: Mass spectrometry-based proteomic analysis confirms expression of EDPCCC and loricrin 2 proteins in toes of the green anole lizard. Figure S3. Amino acid sequence alignment of EDM proteins and evidence for expression of EDM1 in the skin of the common wall lizard.
