# Supplementary material for: Epidermal Differentiation Genes of the Common Wall Lizard Encode Proteins with Extremely Biased Amino Acid Contents
Source: Genes (Basel). 2024 Aug 28;15(9):1136. doi: 10.3390/genes15091136 (PMC11431283; doi:10.3390/genes15091136)
Supplement: Supplementary file 1 [file genes-15-01136-s001.zip › genes-3161762-supplementary.pdf]

Table S1. Abbreviations and full names of EDC genes.

Abbreviation	Full name of gene (tentative)
CBP	Corneous Beta Protein
CBP-G	Corneous Beta Protein Glycine-rich
CRNN	Cornulin
EDCATM	Epidermal Differentiation protein containing the CAT Motif
EDCC	Epidermal Differentiation protein containing Cysteine Cysteine motifs
EDCG	Epidermal Differentiation protein rich in Cysteine and Glycine repeats
EDCML	Epidermal Differentiation protein containing a CCCC Motif Like
EDCP	Epidermal Differentiation protein rich in Cysteine and Proline
EDCRP	Epidermal Differentiation Cysteine-Rich Protein
EDCS	Epidermal Differentiation protein, Cysteine-rich Short
EDCSL	Epidermal Differentiation protein, Cysteine-rich Short Like
EDEPK	Epidermal Differentiation protein rich in glutamic acid (E), Proline and lysine (K)
EDEPT	Epidermal Differentiation protein rich in glutamic acid (E), Proline and Threonine
EDETM	Epidermal Differentiation protein containing an ET Motif
EDGPC	Epidermal Differentiation protein rich in Glycine, Proline and Cysteine
EDGY	Epidermal Differentiation protein rich in Glycine and tyrosine (Y)
EDGGY	Epidermal Differentiation protein rich in a Glycine duplet and tyrosine (Y)
EDH	Epidermal Differentiation protein rich in Histidine
EDHEM	Epidermal Differentiation protein containing a HEM Motif
EDKM	Epidermal Differentiation protein containing a KKLIQQ Motif
EDM	Epidermal Differentiation protein without unique Motifs
EDP	Epidermal Differentiation protein rich in Proline
EDPAML	Epidermal Differentiation protein containing a PA Motif Like
EDPCCC	Epidermal Differentiation protein containing PCCC repeats
EDPCS	Epidermal Differentiation protein rich in Proline, Cysteine and Serine
EDPL	Epidermal Differentiation Proline-rich protein, close to Loricrin
EDPKC	Epidermal Differentiation protein rich in Proline, lysine (K) and Cysteine
EDPQ	Epidermal Differentiation protein rich in Proline and glutamine (Q)
EDPSQ	Epidermal Differentiation protein rich in Proline, Serine and glutamine (Q)
EDQK	Epidermal Differentiation protein containing glutamine (Q) and lysine (K) repeats
EDQL	Epidermal Differentiation protein rich in glutamine (Q), close to Loricrin
EDQM	Epidermal Differentiation protein containing a glutamine (Q) Motif
EDQSG	Epidermal Differentiation protein rich in glutamine (Q), Serine and Glycine
EDSC	Epidermal Differentiation protein rich in Serine and Cysteine
EDSCP	Epidermal Differentiation protein rich in Serine, Cysteine and Proline
EDSPR	Epidermal Differentiation protein Small Proline Rich
EDSPRL	Epidermal Differentiation protein Small Proline-Rich Like
EDSQ	Epidermal Differentiation protein rich in Serine and glutamine (Q)
EDSRWM	Epidermal Differentiation protein containing a SRW Motif
EDWM	Epidermal Differentiation protein containing a WYDP Motif
EDYM	Epidermal Differentiation protein containing Y Motif
LOR	Loricrin
PGLYRP	Peptidoglycan recognition protein
SCFN	Scaffoldin

Table S2. EDC genes of the common wall lizard

Gene	Accession nr.	CDS start	CDS end	Evidence for expression*
S100-A12	NC_041327.1	7826291	7824161	yes
PGLYRP3	NC_041327.1	7801083	7786445	yes
EDKM	NC_041327.1	7770839	7769525	yes
EDPQ3	NC_041327.1	7755522	7755755	yes
EDPQ2	NC_041327.1	7745801	7745592	yes
EDH3	NC_041327.1	7729992	7730537	yes
EDH2	NC_041327.1	7715622	7716008	no
EDH1	NC_041327.1	7707601	7708029	no
EDM5	NC_041327.1	7692264	7692626	yes
EDM4	NC_041327.1	7684007	7683714	yes
EDM3	NC_041327.1	7677046	7677342	yes
EDM2	NC_041327.1	7583536	7583258	yes
EDM1	NC_041327.1	7540441	7540719	yes
EDETM	NC_041327.1	7180462	7180722	yes
EDGY2	NC_041327.1	6931721	6932281	yes
EDGY1	NC_041327.1	6911694	6911281	yes
EDSRWM	NC_041327.1	6882937	6881987	yes
CBP-G2	NC_041327.1	6640953	6642197	no
CBP-G1	NC_041327.1	6566655	6567932	no
CBP1	NC_041327.1	6512532	6511409	yes
EDCC9	NC_041327.1	6502489	6502914	no
EDCC8	NC_041327.1	6491713	6492141	no
EDCC7	NC_041327.1	6477690	6478115	no
EDCC6	NC_041327.1	6468233	6468655	no
EDCC5	NC_041327.1	6453014	6453388	no
EDCC4	NC_041327.1	6448485	6448111	no
EDCC3	NC_041327.1	6438422	6438048	no
EDCC2	NC_041327.1	6432736	6433110	no
EDCC1	NC_041327.1	6426819	6426445	no
EDYM1	NC_041327.1	6413040	6412496	no
EDPL1	NC_041327.1	6379033	6378869	yes
LOR1	NC_041327.1	6366956	6364737	no
LOR2	NC_041327.1	6347841	6346684	yes
LOR3	NC_041327.1	6322689	6321982	yes
LOR4	NC_041327.1	6302103	6301327	yes
EDQL1	NC_041327.1	6291764	6292057	no
EDQL2	NC_041327.1	6287488	6287195	no
EDQL3	NC_041327.1	6276088	6275858	no
EDWM3	NC_041327.1	6263082	6263477	no
EDGPC2	NC_041327.1	6249894	6250220	no
EDGPC1	NC_041327.1	6239994	6240206	no
EDCG2	NC_041327.1	6228493	6228266	yes
EDCG1	NC_041327.1	6217215	6216994	no
EDCRP1	NC_041327.1	6210251	6211612	no
EDCRP2	NC_041327.1	6204209	6203193	no
EDCRP3	NC_041327.1	6188777	6187845	no
EDPCCC16	NC_041327.1	6180150	6179839	no
EDPCCC15	NC_041327.1	6172521	6172117	no
EDCML	NC_041327.1	6164437	6164249	no
EDCSL2	NC_041327.1	6157821	6158279	no
EDCSL1	NC_041327.1	6146286	6146786	yes
EDPCCC14	NC_041327.1	6137623	6137342	no
EDPCCC13	NC_041327.1	6135189	6135431	no
EDPCCC12	NC_041327.1	6130032	6129727	no
EDPCCC11	NC_041327.1	6115959	6116174	no
EDPCCC10	NC_041327.1	6113784	6113551	no
EDPCCC9	NC_041327.1	6100925	6101152	no
EDPCCC8	NC_041327.1	6090664	6090891	no
EDPCCC7	NC_041327.1	6087596	6087372	no

Table S2. EDC genes of the common wall lizard (continued)

Gene	Accession nr.	CDS start	CDS end	Evidence for expression*
EDPCCC6	NC_041327.1	6081312	6081521	no
EDPCCC5	NC_041327.1	6078472	6078245	no
EDPCCC4	NC_041327.1	6072746	6072964	no
EDPCCC3	NC_041327.1	6069638	6069435	no
EDPCCC2	NC_041327.1	6066032	6066244	no
EDPCCC1	NC_041327.1	6058081	6058296	no
EDCS2L	NC_041327.1	6046906	6046334	no
EDHEM	NC_041327.1	6016382	6016041	no
EDPQ1	NC_041327.1	5993825	5993100	no
EDWM1	NC_041327.1	5983311	5982511	yes
EDWM2	NC_041327.1	5975167	5974307	yes
EDQM5	NC_041327.1	5961057	5960824	yes
EDQM4	NC_041327.1	5947111	5946881	yes
EDQM3	NC_041327.1	5937774	5937544	no
EDQM2	NC_041327.1	5900115	5899882	no
EDSC4	NC_041327.1	5895813	5896166	no
EDSC3	NC_041327.1	5879413	5879135	yes
EDQM1	NC_041327.1	5843097	5842864	no
EDSC2	NC_041327.1	5838801	5839154	no
EDSC1	NC_041327.1	5823319	5823032	yes
EDM6	NC_041327.1	5798523	5798798	no
SCFN3	NC_041327.1	2485722	2488802	no
THEM4	NC_041327.1	2460207	2474325	yes
S100A-10_2	NC_041327.1	2443855	2445916	yes
S100A-11_2	NC_041327.1	2419746	2428705	yes
S100A-10_1	NC_041327.1	2407482	2409542	yes
S100A-11_1	NC_041327.1	2356734	2365590	yes
SCFN1	NC_041327.1	2341353	2348731	no
SCFN2	NC_041327.1	2313756	2310571	no
CRNN	NC_041327.1	2295687	2299489	no
EDSPRL2	NC_041327.1	2263766	2263599	yes
EDEPT	NC_041327.1	2250126	2250689	yes
EDSQ	NC_041327.1	2236947	2237723	no
EDPAML	NC_041327.1	2217977	2218633	no
EDCATM	NC_041327.1	2198202	2199806	no
EDSPR2	NC_041327.1	2168697	2168329	no
EDCP	NC_041327.1	2151066	2150503	no
EDSPR1	NC_041327.1	2140389	2139985	no
EDQK	NC_041327.1	2124905	2124702	no
EDPCS	NC_041327.1	2118319	2118990	no
EDSPRL1	NC_041327.1	2093089	2092820	no
EDQSG	NC_041327.1	2086082	2086474	yes
EDP3	NC_041327.1	2077127	2076795	yes
EDPKC	NC_041327.1	2068949	2070034	yes
EDEPK	NC_041327.1	2016665	2016180	yes
EDPSQ	NC_041327.1	1995305	1994499	yes
CBP_last	NC_041327.1	1984092	1984337	no
EDGGY5	NC_041327.1	1951550	1951885	no
EDGGY4	NC_041327.1	1940567	1940178	yes
EDGGY3	NC_041327.1	1924961	1925209	yes
EDGGY2	NC_041327.1	1917909	1917661	no
EDGGY1	NC_041327.1	1911814	1912053	no
EDSCP2	NC_041327.1	1841429	1840500	yes
EDSCP1	NC_041327.1	1833555	1834058	yes

Notes - THEM4 is not a prototypical EDC gene. Introns are not shown. CDS, coding sequence; nr, number. * Evidence for expression corresponds to presence of RNA-seq peaks in the "Genomic regions, transcripts and products" view at NCBI GenBank (www.ncbi.nlm.nih.gov, accessed on 30 July 2024).

A

>Pm_CBP1
MSTSGVKCVTTPCVTSCPDAKVVIHPPPMVLTLPGLSLTTAPNQCLVETQTSCLTNGSELCGDGSSKAIVTKSSGLRAVSGGDTPCCTTTCPDSQVVIQPPPVCITIPGAVLTSY
PNECLIQTSNPCVPSGSQPHAIARSSSVSDCSLTPQRMTRSTSVPCGLNNTPACVTQGPGSKVVIYPPPIEVTIPGPILEIAAEECTVEVYNPCDTAGAITSGEERCAITSGEEGE
VKALTARALSCTKVCGVLGASSSCVSQGPEMKIVIQPPPIEVDLPGPILQVFPEACKVETLTPCAPEPQALCSNSDPATSTALATRTSSLSTIKRPLPDMRRPARPWAEMYSRSIT
PRSILAKTSRFSKYHHGFSTSSYQSSY

>Pm_CBP-G1
MSVGGGGNMCYASCPASTVTIQPPPFVLNIPGPALYCPDQNFGIEQYNPCAGYGGGYGGSGGMGGRAIGSGSGMGIGGGMGGGGGYGGLGGAGGYGGGSMGGRGL
GGLGSGGSLGGGGGYGGIGGGYGGGSMGGRGGGGLGGGSLGGLGGGSMGGSGGIGGSGGRRGSIGGGTGGGRRGSIGGGTGGGRRGSIGGGSGGGRRGSIGGGS
VSGRRGSIGGGSVGGRRGSIGGGSMGGGLGGSGGSVGGGTGGIGGSMGGGGGGRRISGGRRYSGSGGYSSGGYGGSGGYSSGGYGGSGGYSSGGYGGSGGYSSGGY
GGSGGYGSGGYGGSGGYGSGGYGGSGGYGGSGGYGSGGYGGSCGYGMGGYGSGGYGSGGYGGRSYGTRRIIYSGGRRSGYGGGGYSSRSYGGSYGGSLALLPSTACY
TCY

>Pm_CBP-G2
MSVGDGGNMCYASCPASTVTIQPPPFVLNIPGPALYCPDQNFGIEQYNPCAGYGGGYGGSGGMGGRAIGSGSGMGIGGGMGGGGGYGGLGGAGGYGGGSMGGRGL
GGLGAGGSLGGGGGYGGIGGGYGGGSMGGRGGGGLGGGSLGGLGGGSMGGSGGIGGSGGRRGSIGGGTGGGRRGSIGGGSGGGRRDSIGGGSGGGRRGSIGGGS
VSGRRGSIGGGSMGGGLGGSGGSVGGGTGGIGGSLGGGGGGRRISGGRRYSGSGGYSSGGYGGSGGYSSGGYGGSGGYSSGGYGGSGGYSSGGYGGSGGYGSGGYG
GSGGYGSGGYGGSGGYGGSGGYGGSGGYGGSCGYGMGGYGSGGYGSGGYGGRSYGTRRIIYSGGRRSGYGGGGYSSRSYGGSYGGSLALLPSTACYTCY

>Pm_CBP_last
MAFGNCGFTCESQCPPHHVPIQPPPFCITVPGPIMSCANDSCAISCTAPCGYGYGYGAGCGGYGGGWGGFGRGCGGYGGCW

>Pm_EDCATM
MYQTKQPCLPPPIGATKSTDSCTPPCVKTCAVPCPNSCTAKCIEACAVECNPGFVHKPEASCAAPTSPECTGSCAPPCAAASEAPCSSPPCTDVAQCEDASGGPSPSIFEAPIPV
ICLDQCATTGEPPISSGGKTPPPLPSISPDACFATCVDPWAASPDCANACSTKCATPCATKCCDGCDASKGAATITCATKCATSVPCATESTNITPCATKCCDGCDASKGAAAITY
ATKCATSVPCATESTNITPCATKCCDSCGALKGAASVKSATNYATLVPCSTESTSWYPCAIRCCDCSEALKAAACKKCATKCATSVPCATTSSSTTPCATKCCDCCGALKGAATVT
SATECDTPCATKSTSSIPCAVKCYACCAALKAAASNKCATKCATSVPCATGSTSTTSCVAKCYAALKAADAMKCATKCAASVPCATGSTSATPCATKCCDCCSAWKGTAAVPCAT
KCAMSVPCATKSAGTTPCAAKGCEYCGTCVPQVIHTWAMRCGIPCSNACACGSTKGGVSCSASCPCEGSCKK

>Pm_EDCC1
MACCGTSCGSGCGGGSGSGSGGVLVDTGNSSAVHVQPGGCSLTVPGPRLVSHGTTQVSCRCVCASECCPPCGSSGSGNGGSGPGMRVDMGYPGYDVIMPGAYLCSTA
HCGRVSSSCCEPCCGPC

>Pm_EDCC2
MACCGTSCGSGCGGGSGSGSGGVLVDTGNSSAVHVQPGGCSLTVPGPRLVSHGTTQVSCRCVCASECCPPCGSSGSGNGGSGPGMRVDMGYPGYDVIMPGAYLCSTA
HCGRVSSSCCEPCCGPC

>Pm_EDCC3
MACCGTSCGSGCGGGSGSGSGGVLVDTGNTSAVHVQPGGCSLTVPGPRLVSHGSTQVSCRCVCASECCPPCGSSGSGNGGSGPGMRVDMGYPGYDVIMPGAYLCSTA
HCGRVSSSCCDSCCGPC

>Pm_EDCC4
MACCGTSCCGGGYGGGYGGGHGGYGGGGAGAGSGGVLVDTGDTSIVHVQPGGCSLTVPGPRLICHCDPRVSCHCVCASECCPPSCCGSGSYGGGHGGHGGYGPGMR
VDMCYPGYDVIMPAGFLCSTAHCGNVNTSCCECGPC

>Pm_EDCC5
MACCGTSCGGGCGGGYGGGSGGYGGGGAGAGSGGVLVDTGNTSMVHVQPGGCSLTVPGPRLVYHCDPRVSCRTVCASECCPPSCCGSGSFGGGHGGSGPGMRVDM
CYPGYDVIMPAGFLLSTAHCGNVNTSCCEPCCGPC

>Pm_EDCC6
MACCGTSCCGGYGGGYGGGHGGYGGGGAGAGSGGVLVDTGDTSIVHVQPGGCSLTVPGPRLICHCDPRVSCHCVCASECCPPSCCGSGSYGGGHGGHGGYGPGMRV
DMCYPGYDVIMPAGFLCSTAHCGNVNTSCCECGPC

>Pm_EDCC7
MACCGTSCCGGYGGGYGGGHGGYGGGGAGAGSGGVLVDTGDTSIVHVQPGGCSLTVPGPRLICHCDPRVSCHCVCASECCPPSCCGSGSYGGGHGGHGGYGPGMRV
DMCYPGYDVIMPAGFLCSTAHCGNVNTSCCECGPC

>Pm_EDCC8
MACCGTSCGSGCGGGSGSGSGGVLVDTGNTSAVHVQPGGCSLTVPGPRLVSHGTSQVSCRTVCASECCPPCGSSGSGHGGSGPGMRVDMCYPGYDVIMPGGYLCSTAH
CGRVSSSCCEPCCGPC

>Pm_EDCC9
MACCGTSCGSGCGGGSGSGSGGVLVDTGSTSAVHVQPGGCSLTVPGPRLVSHGSSQVSCRCVCASECCPPCGSSGSGHGGSGPGMRVDMCYPGYDVIMPGGYLCSTAH
CGSVSSSCCDSCCGPC

>Pm_EDCG1
MGCCGHSDSCGCHGHGGTRVVYCCVPYSGVQSMRSCCCSCCSPMQSMQSCCAPKSHGCCAQIQQCCVPSKKCC

>Pm_EDCG2
MGCDCCGSSSNSGTIICCVPSGGSNSCCTPCCTPCCAPCCCCAPCCCAKPACCCAPCCCSKSCCTPCCAQVKKCC

>Pm_EDCML
MSCCRCCRGRGCCCCRRPSPRLVYYVSRPNQCCWVTRYSYSSCCAPRAQLPIQQCCPPVKKC

>Pm_EDCP
MSHQCKQPCAPPPCCVKGTTVKCGTASCGATKCATDVCTAPCQTACGDGVKAPAETKCADPCTKCADPCTKCVEPCTKCASPCQSICGGVKAAAPCQGTVCVTPCQGQTC
ASVCATPCQAQTCVPVCASPCQAQTCIPVCIQRCPCGCGKLVLVPQTCCASPCSTVCAVPVQVQPCCSSTCCKPTCCA

>Pm_EDCRP1
MSCSNCCGQSSSSSCGCSQGCCTPCCCNQGSCSQGCGCSSCNQSCCSRGCCCPCQCQQCCCPSCCCPKCCSSCCNQRGCSNCGCSSCGSQCGGSSCCCQKPCCCSKCGC
SPCCCQKPSCCSKCGCSPCCCQKPCCCSKCGCSPCCCQKPSCCSKCGCSPCCCQKPSCCSKCGCSPCCCQKPCCCSKCGCSPCCCQKPSCCSKCGCSPCCCQKPCCCSKCGCS
PCCCQKPCCCSKCGCSPCCCQKPSCCSKCGCNPCCCQKPSCCSKCGCSPCCCQKPSCCSKCGCSPCCCQKPSCCSKCGCSPCCCQKPSCCSKCGCSPCCCPKSSCCSKCGCSP
CCCQKPSCCSKCGCSPCCCQKPSCCSKCGCSPCCCQKPSCCSKCGCSPCCCPKSSCCSKCGCSPCCCSKSSCSVPCCQQVCCKPCCPPCCSTKSPCCSKCGCSPCCCSKGSSQ
QSKSC

>Pm_EDCRP2
MACCQKKSICCSKCGCCPCCCSQTQSCGCSPCCCQKSSCCSKCGCSPCCCQKSSCCSKCGCSPCCCQKSSCCSKCGCSPCCCQKSSCCSKCGCSPCCCQKSSCCSKCGCSPCC
CQKSSCCSKCGCSPCCCQKSSCCSKCGCSPCCCQKSSCCSKCGCSPCCCQKSSCCSKCGCSPCCCQKSSCCSKCGCSPCCCQKSSCCSKCGCSPCCCQKSSCCSKCGCSPCCC
QKSSCCSKCGCSPCCCQKSSCCSKCGCSPCCCQKSSCCSKCGCSPCCCSQSCCCSPCCCHQCCCKPCCGSCCQCRPCCCSPCCCCNQGCCSPCCCGSNSCCSSGCNSFQNKC

>Pm_EDCRP3
MACCQNKSQCCSKCGCSPCCCSQGQSCSKCGSSPCCCHKSKCCSKCGCSPCCCQKSSCCSKCGCSPCCCQKSSCCSKCGCSPCCCHKSKCCSKCGCSPCCCSQRQSCSKCGC
SPCCCHKSKCCSKCGCSPCCCQKSSCCSKCGCSPCCCQKSSCCSKCGCSPCCCHKSKCCSKCGCSPCCCSQRQSCSKCGCSPCCCHKAKCCSTCGCSPCCCSQSCCSRCGCSP
CCCRKLTCCSRCGCSPCCCSSQSRCCCPRCCNQSCCSPCCCSRCRRSPCCCCNQCGCSPCCCCNQSCCVPCCCHQTCCSTYQYKC

>Pm_EDCS2L
MGCNDCCSSSCGTALLCCAVPCCGQSCCSPCCGQSCCGQSCCSPCCGQSCCGQSCCSPCCCQSCCSPCCGQSCCSPCCGQSCCSPCCGQSCCSPCCGQSCCGQSCCSPCC
GQSCCSPCCGQSCCSPCSSCKSCCSPCCGQSCCGQSCCSPCCGQSCCSPCSSCKSCCSPCCSQTCCAPCCTTKCCGTKKC

>Pm_EDCSL1
MSCCSCCGCSPCCCRYCPCCCCCPCCPCCCPCCCCPCCGSGSSGSSSGGSSCCCPRQGSSSSGGSSCCCPRQGGSSSGGSSCCCPRQGGSSSGSSSCCYPSQGGCSSGGSS
CCCPRQGSSSSGGSSCCYPSQGGSSSGGSSCCYPSQGGSSSGGSSCCYPRQRGWC

>Pm_EDCSL2
MGCNDCCAVPCCGQSCCGQSCCSPCCCQTCCSPCCCVPCCSQSCCSPCCSQSCCGQSCCGSSKGCCQSCCGSSKGCGQSCCGSSKGCGQSCCGSSKGCCQSSCGSSKGC
CQSSCGSSKGCGQSCCSIPCCQTCCTPCCPPCCSTKGCCSKKC

>Pm_EDEPT
MAYQCRQPCLPPPIGVPQCAIDSSGACAPTCGDPCADPCSAASYQSKQPCLPPPVCTPLCQAESAPAPTSTCSACEELCAAPSVEVSSAPCELPTIPANADPCAPPCATLCSSW
ECSGASAAPSREAVESYVVPGSIVSLNPSGHVSVKPGPEVEVGSCESVFLNSYTPTFPKKQQGVFAALGQQRD

>Pm_EDETM
MVFPGELCGYNVSCSTSLPSTTLLLQPPAVVAETKLTDLEILIPEPPPCEIPECGFDPCHLPPWYCHLPPEPPIIFATFSTCLHEA

>Pm_EDM1
MSFLNDSMFLEDFYEPEMDAHSSLDSFSYQYPGCYPQRPQFPGRSWGCPRGYRPITGCIPCYPGLPCPPGYRVIRICVPFGRASYQGKGWPC

>Pm_EDM2
MSFLNDSMLLEDFYEPEMDAHTSLDSFSYQYPGCYPQRPQFPGRSWGCPRGYRPITGCIPCYPGLPCPPGYRVIRICVPFGRASYQGKGWPC

>Pm_EDM3
MSFLNDSMLLEDFYEPEMDAFSSFDSLSYQYPGCYPQRPQFPGRIWPCPRGYRPITGCIPCYPGLPCPPGYRVIRVCVPFGRASYQGKGWPCKSETQH

>Pm_EDM4
MSLLNNGILLEDFSNPEDDSFCQYPGCYPPYQRPGCYDPCGSKYGPGFGFPCPPGYRPIKACVPFYPRPSWPCPPGTYPRKVCVPYRRYYPGQGWPC

>Pm_EDM5
MSLLNNGIQLEDFSKPEADSFSSLDSFCCDSPCYYPQNFPCSPGCSLRIVFIPRFGQRCPQGYAPVEVHIPICASVGPCPIPYRRIRGCLPIRPGAPCPPGFQPIRACVRNQFPGP
CYPC

>Pm_EDM6
MSLLNDGILMEDFYDPEMEGLDSFYQYPYCYRPRQRSCYYPRGYGYGYGYGGGYGSKWRPGYTWQYPQYCPPQYYGPGRWPCYYPGKRWPC

>Pm_EDGPC1
MGNGGSSCCDCGGNSGNDTMIVCTPSSGCRPCCTPCCCAPMCCSPCCSPCCCCHPSCCTPCCSQMCCRKC

>Pm_EDGPC2
MSCCGCCGGCCGGSGSGSTIIYCPMPMAPQPCYAPQCIPVQSYYPSMQSCCQSLQSACAPGKACCQSLQSACAPAKACCQSLQSACAPVKSKGGAIPLQQSLGSAKIC

>Pm_EDGY1
MLYRQSSCYQPCYQPCYQPCGYGGGTGYSSGTSCGYGYSSGCGGYGSGGYGYGPACMPPCSYGYRYSRRGGCYDPSAYRLGYGARLNSPSRRYGGSYRGSCGGSYGGGY

GGCYGGGYGGGYGGGYGGGYGGSCGWPC

>Pm_EDGY2
MSTFKHQSQCDPGYCRPTTSSSSCYGGSGHGYSSGHGYSSGSCSGGSGRRIRYSSGSCYGGYGGYGQGGYGRGYSYGSGCYQPLSYGGFGSRGWGYGGQGCGRICYQPI
SYGGWGYGGRGCSPQGGYGGLGYGGYGYGGCGYGGLYGRGSSCYQPCYGYGSRRGSFSYPIVRRNSICGPCGPCGPC

>Pm_EDGGY1
MTYSCSVACRPWRHQSCYEPCGYGYGYGGWCSSRYCAPRCYEPCGYGYGGYGGYGGYGGYGDWCGSRWGYPYSRWGCGW

>Pm_EDGGY2
MTYSCSVACRPWRHQSCYEPYGYGYGYGGWCSSRYCAPRCYEPCGYGYGGYGGYGSYGCYGGYGDCCGSRWGYPYSRWGCGW

>Pm_EDGGY3
MTYYGGYCRPYGYSSCSTSCYDPCCYGSGYGHWYGSTTCRTGCYDPCRYGGYSGYGCYGGSGCYGAYGSKGCYPYSRWGCGW

>Pm_EDGGY4
MTCGYGNCCSPCGSGGWGYGGYGGNCCGNRCGSGYGGYGSYGGYGGNCCSNRCGGYGGYGGYGGYGGYGSNCCGNRCCGGYGGYGSYGGYGGYGGNCCGNRCG
GYGGYGGYGGYGGNCCSSRCCGGWGW

>Pm_EDGGY5
MSNYGTYGLSSCSGNQSGREPSSQGSGHWFGSTTSRPRCYEPSSYGSYASSGGFGGCGSCGGGYGGSGRMGGHSGFGSFASFGGCGGQGGYGGWGNPRPCASSPWG
SGWGN

>Pm_EDH1
MTPGNHHRYSTFLNSPNNTTGESSKVPGVDSLHCCPSHWHLWHHHPYSYPCGPGWGYGCGSGWVPDCGYGPGCEPCYPYGGYGYPHAVHCYPLGGHCYPQGGHCYP
PGGHCYPHGGHCYPPGGHCYPPGGYCYPPGGYCYPC

>Pm_EDH2
MTPGNHHRYSTFLNSPNNTTGESSKVPGVDSLHCCPSHWHLWHHHPCSYPCGPGWGYGCGSGWGPDCGYGPGCEPCYPYGGYCYPHAVHCYPPGGYCYPPGGHCYP
PGGHCYPPGGHCYPPGGYCYPC

>Pm_EDH3
MTPGNHHHYSTFLNSPCNTTGESSKVPGVDSFHCWPCHWHLWHHHPYYYPCGPGWGYGCGSGWGPDCGQGYGPGYEPCYPYGGYGYPHAWHCYPKKVKVKGCDPS
GGCCNPSGGCCCPKKVKVKWGNPYGGCCNPSGGCCYPKKVKVKGCNPYGGCCNPSGGYCYPPGGYCYPQGASCYPC

>Pm_EDHEM
MAYDKHSAVSCADEKHGARLIVQAQGGESGQGGVDNGTSSNLGDERTGHSFTIPQLDRGGNEISPRHNDTAASGSPASDNLIVLNSFTVSYRWSPMLKYCKPKELGRGPIL
LC

>Pm_EDP3
MSQQCKQPTSCPPTFCGSASSGCGKTVQVPACQGQVEPQCPGNPCQGQVEPQCPGNPCQGQVEPQCPGNPCHGSGGGSQGHCQLAKDGGSHHQHHQHHQHHQD
SQQAKQC

>Pm_EDPAML
MTHQCKLPATAPPSCVKGVAVCAAPQATKGVAVCGAAPSPCSACAATPVTVCGIPQQTACGAKTISVCGIPQQTSCGAKTISVCGIPQQTSCGAKTISVCGVPQQTACGAPTL
TVCGLPQQTTCGASTLTVCGLPQQTTCCGAPALTVCGLPQQTTCGAQATLVPSCVSSPLVISSGCGCGGVPLSSCVPVTLNLVQPTCVAAAPCTSTSCCAAPGCV

>Pm_EDPCCC1
MSCCCKCGCSPCCCKQCCCSPCCGCGCGCCKQCCCSPCCCKQCCCSPCCGSGCCGSSCCGCKCCYPKQCKC

>Pm_EDPCCC2
MSCCSNCCCSPCCCKCCCCPCKCCCSPCCCCTPCCCCCSPCCCNSCGCGSCGCGSCGCGSCCCYPKQGCC

>Pm_EDPCCC3
MSCCCQCGCSPCCCKQCCCSPCCGCGCGCCKQCCCSPCCCKQCCCSPCCCCSPCCGSGCCGSSCCGCKCCYPKQCKC

>Pm_EDPCCC4
MSCCSNCFCSPCCCKCCCCPCCCCTPSCCCTPCCCCCSPCCCNSCGCGSCGCGCGSCGCGSCCCYPKQGCCC

>Pm_EDPCCC5
MSCCCQCGCSPCCCKQCCCSPCCGCGCCKQCCCSPCCCKQCCCSPCCCSSPCCGSGCCGSSCCGCKCCYPKQCKC

>Pm_EDPCCC6
MSCCSNCCCSPCCCKCCCCPCKCCCSPCCCCVPCCCCSPCCCNSCGCGSCGCGCCGCGSCCCYPKQGCC

>Pm_EDPCCC7
MSCCSNCCCSPCCCKCCCCPCKCCCSPCCCCTPCCCCCSPCCCNSCGCGCGCGSCGCGSCGCGSCCCYPKQGCC

>Pm_EDPCCC8
MSCCCQCGCSPCCCKQCCCSPCCGCGCCKQCCCSPCCCKQCCCSPCCCSSPCCGSGCCGSSCCGCKCCYPKQCKC

>Pm_EDPCCC9
MSCCCQCGCSPCCCKQCCCSPCCGCGCCKQCCCSPCCCKQCCCSPCCCGSPCCGSGCCGSSCCGCKCCYPKQCKC

>Pm_EDPCCC10
MSCCCQCGCSPCCCKQCCCSPCCGCGCGCCKQCCCSPCCCKQCCCSPCCCCSPCCGSGCCGSSCCGCKCCYPKQCKC

>Pm_EDPCCC11
MSCCSNCCCSPCCCKCCCCPCCCCSPSCCCVPCCCSSPCCCNSCGCGSCGCGCGSCGCGSCCCYPKQGCCC

>Pm_EDPCCC12
MSCCSNCCSPCCSPCCCCSPCCCRSCGCGSGCCGSCGCGCGGCGSCGCGCGGCGSCGCGSGCCGSCGCGSGCCGPCCGCGSGCCGSCGCGSGCCYPKQGCC

>Pm_EDPCCC13
MSCCSKCGCSPCCCKQCCCSPCCGCGCCKQCCCSPCCGCGCCKQCCCSPCCCKQCCCSPCCSSGCCGSSCCGCYPKQCKC

>Pm_EDPCCC14
MSCSSNCCCCPCSCKCCCCPCCCKCCSCPCCCCCPMLLLLPSCGSGSSGSSGSCGSSGSCGSSRGSCGSCRSCGSQGSGGSGSSCCYPKQCWC

>Pm_EDPCCC15
MSCCGGQSSRCSKCGCSPCCCQSRQVCSRCGCSPCCCHKRSCCSRCGCSPCCCQSRQVCSRCGCSPCCHRRSCCSRCGCSPCCCHRRQACSRCGCSPCCCHRRSCCSRCG
CSPCCCSSRGYCNQSRYTCQQYKF

>Pm_EDPCCC16
MSCCGGQSSHCSKCGCSPCCCQSRQVCSGCGCSPCCCHKRSCCSRCGRSPCCCQSRQVCSRCGCSPCCCHRRSCCSRCGCSPCCCSSRGYCNQSRYTCQQYKY

>Pm_EDPCS
MEFLHSYPELIDVSGPILLDTGTTVLIKQSPECLPTKAPYCIQPSHAQSIHIPPSKYFDHCAKSCHPRCLTPCKSICGPQSCGIGPSKCVPIYGGPQCVSPCYPLCRRPYLPQCLPPC
RPICGYPCQPQCGPCSPICVRPQCVSPCYPIYRNPCLSPCLSPCRPICGYPCAPQCVYPRPGICLQPSYQYPQISCGYPPCGYPCCSQCGQPCPCDSLDLFYEGY

>Pm_EDPKC
MASFNNQQQRKQATTLPPALCKTAPEPLPCPQPTPCPQEKPPCKETPVVVVPTPCPRPTPCQEKPPCKEPPVVICPTPCPQPTPCQEKPPCKEPPVVICPTPCPQPTPCQEKP
PCKEPPVVVVPTPCPQPTPCPQQKQPCKEPPVEICPTPCPQPTPCEEKPPCKEPVVVVPTPCPQPTPCPQQKQPCKEPPVEICPTPCPQPTPCEEKPPCKEPVVVVPTPCPQP
TPCPQQKQPCKEPPAVICPTPCPQPTPCEEKPPCKEPVVVVPTPCPQPTPCQEKPPCKEPPVVVVPTPCPQPTPCQGKPPCKETPVVVVPTPCPEKQPPCKEPATVPCCPPEA
KCPPPCDQQQKKQPCQWPHQQK

>Pm_EDPL1
MSSQQSQCPTNPQPCQDNPQKCPPKQDADCTPPKTCSPPQDQQKKAPPSCPQNPK

>Pm_EDPQ1
MYPPQYRDDQDSCDDYGASVCSEPSCAPSGASYDPCHSVSSSSAVDCSGTSSQQYCPPAQKYCPPAQKYCPPAQKYCPPQQKYCPPAQKYCPPAQKYCPPQQKYCPPAQKY
CPPAQKYCPPQQKYCPPAQKYCPPAQKYCPPAQKYCPPVQKYCPPQQKYCPPAQKYCPPVPKCCPPQQKYCPPVQRYCPPVQRYCPPVQQYCPPAQTCCPPPQQTYTPVGE
PEICQIQQVCQAPPHLLKK

>Pm_EDPQ2
MSQQQQQCKQPCQVPPQCCPPQQDCLPPQQKCPPQKDCLPPQQKCPPQQDCLPPQQKCPPQQTQDPKCC

>Pm_EDPQ3
MSYQSQQCKQTCPPPPQCKKAPQKCCPPPQQCCPPPQKCCPPPQQCCPPQQKCCPPPQQCCPPPQQCCPPKQDPPCC

>Pm_EDPSQ
MYSYEQQCKQPCLPPPICQQNTGIKGTKGYRPQSQQPYISPCSPQWQGSCTPGYLQSPYAPQSPQSYAPEYIQPCSPQSCYEPQSTQSCSPQTYGTPCIPVCYPQGQKCAPP
QSIQPCYPPQQKSLSSSQCTQQTCCPPCKSATFSCQQSCATQTTQCSPACATKGDWLCTTKGGQQYVSQQCITKGGPSSQQYSSQSSFTKGSSGYASKGQQQWSSGVTKG
GPVYSTKTSQKYSRKGGQSFGPQQCKTQVKISSKGQKYCSAGKWPF

>Pm_EDQK
MSGQEQQQKKHSYVPPVVHHPPPQQKPPGCGTETHHVPKPPSCGKPKPDEHHHQQQEEQKPKHKSCH

>Pm_EDQL1
MCSRNDRGCHNKPQDSCHNKPQDSCHRGGSSCHEDRGCQQQPPVVIPAPCIPPVVIPAQCPKQPPVVVPPPCIPPVVIPPSCQQQTKQTPQWPQQQK

>Pm_EDQL2
MCSRNDRGCHNKPQDSCHNKPQDSCHRGGSSCHEDRGCQQQPPVVVPAPCIPPVVIPAQCPKQPPVVVPPPCIPPVVIPPACQQQTKQTPQWPQQQK

>Pm_EDQL3
MCSRSDRGCRNKPQDSCQRQPPVVTPPPCNPPQVVPPMCPKQPPVVITPPCIPPVVIPPPCQQQTKQTPQWPGQQK

>Pm_EDQM1
MCSHQDKDQCYKQEKEEGSCCGGRRSSTSEDRGSCCLGRRNSGGCHGNRGSSGCHGKQQEQQQQQQQQQVFQVPSQK

>Pm_EDQM2
MCSRQDKDQCYKQEKEEGSCCGGRRSSTSEDRGSCCLGRRNSGGCHGNRGSSGCHGKQQEQQQQQQQQQVFQVPSQK

>Pm_EDQM3
MCSRQDKDQCYKQEKEEGSCCGGRRSSTSEDRGSCCLGRRNSGGCHGNRSSSGCHGKQQEQQQQQQVFQVPSQQLK

>Pm_EDQM4
MCSRQDKDQCYKQEKEEGSCCGGRRSSTSEDRGSCCLGRRNSGGCHGNRSSSGCHGKQQEQQQQQQVFQVPSQQLK

>Pm_EDQM5
MCSRQDKDQCYKQEKEEGSCCGGRRSSTSEDRGSCCLGRRNSGGCHGNRGSSGCHGKQQEQQQQQQVFQVPSQKLK

>Pm_EDQSG
MSQQHKQQCKQPPVCSPGPCGNPAKSNGPQWHGSTCSHCHSSTGSYGQGSGGYDCQNSTGSYGQGSGGYVCHNSTGSYGQGSGGYDCHSSTGSHWQGPGGSLSQE
PGQWPSGKDCDDQVPACHPHGTQSY

>Pm_EDSC1
MSQQQQRGSCCCCGGRGCGCCGGSRSGGGCCGGSRSVVSSQQSQSSGGCCGGSSGSSGGGCCGGSRGGGCCGGSSGGGCCGGQQSQQKVFPQKLK

>Pm_EDSC2
MSQQRQQQGGCCGGGGGGHGGGGCCGGGGGGSSQQSQRSGGCCRGGGGGGGGGGGGCCGGRGGGSSQQSQSSGGCCGGRSSGQSSGGSSGGCCGGGSSGGS
SGGCGGGGSSQQMKMK

>Pm_EDSC3
MSQQQQRGSCCCCGGRGCGCCGGSRSGGGCCGGSRSVVYSQQSQSSGGCCGGSSGSSGGGCCGGSRGGGCCGGSSSGGCCGGQQSQQKVFPR

>Pm_EDSC4
MSQQRQQQGGCCGGGGGGHGGGGCCGGGGGGSSQQSQRSGGCCRGGGGGGGGGGGGCCGGRGGGSSQQSQSSGGCCGGRSSGQSSGGSSGGCCGGGSSGGS
SGGCGGGGSSQQMKMK

>Pm_EDSCP1
MVSCCLPSCAISCGPIYPVSSYQPCCSPSCVSPCGPIYPVSSCHQPCSYPCGPIAPLCIQPSCQPCCSPSCVIPCGPIYPASSCHQPCHQTCSYPCGPIAPLCIQPSCQSSCSPSCV
FPCGPIYPVSSCQPCCSPSCVIPSGPIYPPSSCHSSCIPISICNPCCTPC

>Pm_EDSCP2
MVSCYSGPCCTVPCGQSCCAPSYTFPLCGHPCGVKSCSVPSCTTPCGPIYPVSSCQSSCVPSCSIPCGPVHQIISCQPSHVQSCAVPCGPIYPVSSCQPQSCAVPCGPIYPASSC
QPQCVQPSVVKSCSVPSCVVPCGPTYPVSSCQPSCSIPCGPVHQIISCQPSHVQSCAIPCGPIYPVSSCQPQCVQPSVVKSCSVPSCVVPCGPTYPVSSCQPSCSIPCGPVHQII
SCQPSHVQSCAVPCGPTYSVSSCQPCSVPVCSIPCGPVHQVSICHQSCLPSCVIPSGPIYPVSSCQPCAQSCIPCYPPC

>Pm_EDSPR1
MSCHQHQCKQACLPPPCVKSCQPCAPQQCVKTCCPPCGPKCGPACGSGCGPKCAPACGAGCGPKCTPACGSGCGPKCAPACGSGCGPKCAPACGSGCGPKCAPTCAPAC
APPCAPQCVKKCATKCVEPCTPKCK

>Pm_EDSPR2
MSCHQHQCKQACLPPPCVKSCQPCAPQQCVKTCCPPYGPKCGPACGSGCGPKCGPACGSGCGPKCAPACGSGCGPKCAPACGSSCGPKCAPTCAPVCAPPCAPQCVKKC
ATKCVEPCTPKCK

>Pm_EDSPRL1
MACPFQQCKQPCLPPPICGQKGSVKCGDVCPCSASCGPSIVQQSPGQSVTVCSPPCSGPCAKIGGDPCAPVCAPPCTPKCGDVCAPKCVDPCGPSVCAPPCAQVVAPCAA
KCPDPCQSVCAPPQCIPVAVGSCSSKCVPVDPCAKQGPC

>Pm_EDSPRL2
MSQCKQGCKAPPTKCPPKTAQCCPPAVKCPPKTVPCCPPAVKCPPKTKQPPKGCC

>Pm_EDSQ
MSYQCKQPCLPPPSCVKTKSATVCATPGGAVCVTPSQSSCADACTPKCATVCATPGGSICVTPQQPQCATVCQGPCGSISVTPLQNQGPTICATPGGSVCVSQGQGQCSTV
CQGPCGSVCVTPQQPQCATVCQGPCGSVCVTPQQSSGSCATVCTSSGGSICMAPSKGQCASVCQDPCGAVCVAPIPAPTGGACATVCQDACGNVSVTPCATKCADSCSG
AKTCATKCGNPCVTVCSDQTCVKACPSINMGQCNVKKC

>Pm_EDSRWM
MIGISLLSNYPPGGLQMIHTPQSISGSGPSISHTSCYRPYHSGSSCYRPQGYGYVSGYGGSCYNPCGYSRQSSYGGSCYRPQGYGYVSGGSCYDPCGYSCQSSYGGSCYRPQ
GYGYVSGSGGSCYDPCGYSCQSSYGGSCYRPQGYGYISGKSCYGPRGYKSLSGYSGSCYEPCVHSPLYSYSKSCSGSHGYGYSSGYGGSCCDPCYGGSGYKPQGCGYLSGQ
GGSSCYKPRKYSSVCGYGGSCHEPRGYGYRCGYGDSSCDPCYAPSCHQPRYAYRRRYSCRSTSSDPCYYGSGSSYPSSHWRHDSGSSIGCGPC

>Pm_EDWM1
MCEEGCNDRVIYSSGREPWFNLNSTWYGPAGSWLDNRRKPFRYVVNTACVTGCNRRDDVPRRGGHNYRCYGYRRSTCRQGGNPRVTCCVHNPSGGPRDYWGRPIGDS
CDGNTGGHYSNEESFCCGSGGGCGGGSAGGCGGGQGGACAQPVASSGGCGGGRGVCSEPGCGRGRAVCAEPGCGRSSRGLQTSGGAGCAGSSGGCGGGRGVCSEP
GCGRSSRRPQTSGGSVCSESSVRSSGRCGRGRGVCFEPGCGGGRGVCSEPGCRS

>Pm_EDWM2
MCEERCNDRVIYSSGREPWFNLNSTWYDPAGSWLDNRRKPFRYVVNTACVTGCNRRDDVPRRGGHNYRCYGYRRSTCRQGGNPRVTCCVHNPSGGPRDYWGRPIGDS
CDGNTGGHYSNEESFCCGSGGGCGGGSAGGCGGGQGGACAQPVASSGGCGGGRGVCSEPGCGRGRAVCAEPGCGRSSRGLQTSGGAGCAGSSGRCGGGRGVCSEP
GCGRGRAVCSEPGCRRSSRGLQTSGGAGCSGSSGGCGGGRGVCSEPGCGRGRGVCFEPGCGGGRGVCSEPGCRS

>Pm_EDWM3
MTCSSGRESYFNLNSVWYDPAGSWLDTRRTPFCYGNNTCCMTCCDKDLPIRGGHNYRCYGSRCLCTGTATEYCCQRGPCCVRRPSGGCRDYWGRPAGECQNGGGLACA
ESCHGGCCSTEGPVCEEECNSRA

>Pm_EDYM1
MAFYSHHCRIQHSFPTSGPCVNPGPQCFAMKHSTCIKNHPPFAARCPEPCDLKCKIQCPEPCAPKSPVPAEAKGAEACDAACAEKSSSPRMSPSTKPCHETCNTKDPLQHHP
ERCNLSGPFPGPFPGPFPRLVQSGMPKHPMSCGPNFGAQRGFPSSSRCFPPRNMYQYTASKTYKSCYAK

>Pm_LOR1
MSSMQQKQGGGSCCGGGGGGGGGGGGGGGGGGGSCCGSSGGGSISYQGQGGSSGCCCCCGGGGGGGGSGGGGGQKIIIVSGGGGGGGQGCCGGGSSGGGGSIG
GQSSGCCIGGGSGGGQGGGMSQQKIPYMSGGSGGGGGICCGGGSSGGGGGSGGVKIIGGGSGGGGGGGGGGGGSSGGIKVIGGGSGGGGGGGGGGGGGGGSSGGI
KIIGGGSGGGGGGGGGGGGGGSGGVKIIGGGSGGGGGGGGGGGGGGSGGVKIIGGGSGGGGGGGGGGSSGGIKIIGGGSGGGGGGGGGGGGSSGGVKIIGGGSGGGGGG
GGGSSGGIKIIGGGSGGGGGGGGGGGSSGGVKIIGSGGGQSSGGQGSGGQSMGGGSGGGGMGGQSIGCGGQSSGSSIGGGSGGGGGSGGQTIIVPCGGSSGGGGG
QSSGGYIGGGGGGSGGQSSGGCIGGGSGGGGGGSGGQTIIVPCGGSSGGGQSSGGYIGGGSGGQSSGGCIGGGGGGSGGQSSGGYIGGGSGGGGGGSGGQTIIVPC
GGSSGGGQSSGGYIGGGSGGQSSGSCIGGGSGGGGSGGQTVIVPCGGSSGGGGGQSSGGYIGGGSGGGGGGSGGQTVIVPCGGSSGGGGGQSSGGYIGGGSGGGSG
GQSSGGCIGGGSGGGGGSGGQTIVVPCGGSSGGGGGGGQSSGCSIGGGSGGGSGGQTIIVSGGSSGGGGQGSGCCIGGGSSGGSMQTKQPVCLPPGLAQTKQSSQW
PPSHK

>Pm_LOR2
MSQQRQSGGCCGGSGGGGGGGGGGCCSGGSSGGGGGGQSQSSSCCGRGGGGGGGQSQSSSCCGRGGGGSGGCCGGGGGGGMMQIRGGSGGGGGGGCCCCS
GGGGGGGQSGGIIIIPSGGGQSSGCCGGGGYGGGQQRIPIVIGGGSGGVCCGASGGGAVCCGSSGGGGGGESSGCCGGGGSGGGMGQQKIPIVIGGGSSGGSGGAVS
CSSSGGGGGGGAVCCSSSGGGSSGGVKVLGGGSGGGGGGGGGGSGGKTIIIGGGSGGGSGGGVCCSSSGGSGGGGAVCCSSSGGGGSGGVKVIGGGSGGGGGGG
GSGGKTIVVSGGSSGGGGGGGGGGAVCCSSGSGGAVCCSSGGSGGGSSQTKCPIVVPVCIGQTKQPCSMPSIK

>Pm_LOR3
MSKQRQSRGGCCCCGSSRGGCCCCSGGGGYGGGGQSGGVIVIPSGGGYGGGYGGGQQKIPMVVGGGSGGVCCGASSGGAVCCGSSGGGGQSSGCCVGGGMGGGY
GGGYGYGQQKIPIVVGGGSGGVCCGASGGRGGAVCCSSSGSGGAVCCSSSGGGSSGGVKVIGGGYGGGGYSGGSGKTIVVPGGSSGGGSGGAVCCSSGSGGAVCCSSS
GSGGGYSQTKCPIVVPPCIGKTK

>Pm_LOR4
MSQQKQSGGSCCCCGGSGGGCCCCSGGGGGGGGCGRGGYGGQSGGVIVIPSGGGYGGGQQKIPMVVGGGSGGVCCGASSGGAMCCGSSGGGGQSSGCCVGGG
MGGGYGGGYGGGQQKIPIVVGGGSGGVCCGASGGSGGAVCCSSSGGSGSGGAVCCSSSGGGSSGGVKVIGGGSSGGCGGSGKTIVVPGGSSGGGSGGAVCCSSGSG
GAVCCSSGSGGAVCCSSGGSGGGSSQTKCPIVVPPCIGQTKQVCPLPSIK

B

>Pm_Crnn
MSQLLGNIHSIIGAFNKYAKDDGECATLSKGELKSLIQKEFAEVIVNPQDPETIETMLQLLDKDCDGKVDFEEFTVLVFKVAKACYKMEHECGVTAEGQSRRGGSSARRQETSA
LSKGSTQQPAQEPEKVSEPESQCSSQEEKQESPKSETLSDRRSNEPLKQERGSRFSQKPSGACPTQREREEGQRGEKEPSCQGFRREVQHCSKPSQRKPQIVAEEISRQSCQE
PDTPDECQAQGTQQSTVQKPSQPCQMGEQGHREPQEQEQAAQQPTPHQKELPRQTQEQLKREQDVVKPKSQESQKAVRRPAQRGVEEPQAPEQKSTSQPCRDRQEEQ
PSLGCGKERTSTSEQHSTCCQEQEPQRGEWSMQQQECKPQTRGKGAVRRLVEEYQEAKDYSCQQPTKEVQEQEQAQSCKPDGGILTRGKQSTHQQECPSQGHEQALTG
QEEHESQTPEEATTGCHERQPLTTVRHQPHQDVQEPQRSGKNHYRRHECQTLTPEQVVSPQEDTKPQTSEKEACGEPTESTEQTTTGCHEGQRLTRRQHEPYPEESKPQNT
RQSQYRSHECSPLTPEEGPFPQERTTSQKQEREEAEGSEKATTARCECPSLRRRQPCPEESKPQKPGQSQHRSHACQPWTPKQVPSAQEMSQTQEREASCELPEAPEKATSR
GRPSEQCQPHVEERKPKKPGQSQHRCQESQPLVQEQALSPKEETKPQVSKREASCESVEIPEKATSGGRTCQPSPSERCQSHSEEQKPQRPRQSQHHCQASRPLTQEQALS
PKEETKPQVSKREASRESVETPRKATSDGHACQSSTSEQSQTCPEERKHQKPRQSKDQHECQSSETERGISPQEETNSCEQEQESSSQEAEAPERATTGRWKGQSSSTRHQP
HPEEGKPQNSGQIQYCPHECRPLTPEQATSPQEETKPQTSEREGSCEPTQSPASQGPIDSLARQPLRPWPQQQRRALQFPSPWSPKH

>Pm_Scfn1
MPYLLGSICTIIGVFYKYAKRHGDCSTLNRREMKRLILKEFGEVIKNPSDPETVEMTFQLLDINGDSLVDFNEYLLLIFRVAKACYSHLQPRECLLQQGEARRALREREARGEGRD
DRHLREDEREGSYGRERRDSDRTRLHQLEAVARDEGRRESFPREAVARGVERSRHARDQELRDDRGRDRPSHEPQDSENEHEDWPRKSEEQADVGTRQQPLGDEPRRSD
AGRRQLRRREPEMEEDHRRQSRERVQREDGERRPLLRDPRLREGGESERYSQEATQRDEDERRHQARETERQLEEEEDLRRPHVSGPRIDEESHNRRRAVQEIRSDYGRTR
PSREPREREDARRDDYVREAERRSSRRDQRVSDARRDQRSDREPVDYDDERHSFPREGERRSWSRDAEQEQRRSQMPEEEYRERPRSSDPDYRVDEQRRRYTAQSREAEL
HEPDVRESERRRRQPVDQDEWNRPYPRESTTREELEDQRGSRDPERRERQWSGLHPYERKPTDGERRRPQSRESELREGDRRRSQRHAQTPEDGRRREQYHQDDAQEEE
NLRRRRPLSGDLEPRDYESCRPQRRERDPRSDGQRRQQYYDPEPLDRDVERRMPEAPESALREGSRGRQQYSDPDTQERGSRRRPQSRYSESREDERRRPEEADFSDDERE
RPQARQSLSRPREDNRCYPSVGSRRECDRGRRQEYSGQRRNVDQSGDEQERRAPLRDTDAADDEQWRSQSRDIEPRDGEQQRRTQSRDTDGQRRSQRCNIDQSDDEQ
ERRTPLRDTDAEDAEQRRSQSRDTEPRDSEQQRRTQSRDTDGQRRSQRRTVDQSDDEQERRAPLRDTDAADDEQRRSQSRDIEPRDSEQQRRTQSRDTNGQRRSQRRTV
DQSRDEQERRAPLRDIDAEDSEQRRSQSRDIEPRDSEQRRRTQSRDTDGQRRSQRRTVDQSDDEQERRTPLRDIDTEDAEQRRPQSRDTEPRDSEQRRRTQSRDTDGQRR
NVDQSRDEQERRTPLRDIDAEDTEQRRSQSRDIEPRDSEQQRRTQSRDTDGQRRSQRRTVDQSRDEQERRVPLRDIDAEDAEQRRPQSRDTEPRDSEQRRRTQSRDTDGQ
RRNVDQSRDEQERRTPLRDIDAEDTEQRRSQSRDIEPRDSEQQRRTQSRDTDGQRCTVDQSRDEQERRAPLRDIDAEDAEQRRSQSRDTEPRDREQRRRTQSRDTDGQRR
NVDQSRDEQERRTPLRDIDAEDTEQRRSQPRDIEPRDSEQQRRTQSRDTDGQRRSQRRTVDQSRDEQERRVPLRDIDAEDAEQRRSQSRDTEPRDSEQRRSTQSRDTEQR
DSQQRRTQSHYSDTTDGEQRRTRSGDPRFDGQQRRAPSQDTDATDIDRRQTQARGSDPREGEQQRRPQSRGAEPADVDQKQTPTRDADPRNDEQQRRVVSRDTEQSD
GEQRQTQLHSVDARIAAARDGRQQRRTQTRDVDSRDAQQQRRMQSSDTEPTDGDQRQTQTSEAAPRERGQQRQTESRDTEQGASREEPRYSEPKDNVQRRQAVPETQ
VAQPSSVRQAEPLAQDVEGSRAPPREVAPSADTARRPRPQEQEAAERVLGRQRPAESGETQQRPSERERAQSREPQRSDGEQGRARPGGPVPRQSTRSLCGPERSRAERS
QPRQPEPQEGDSRRQQTQDPGSTDGKTSQPREPQAADRQRGRPNTEPRAEGKSEGREGAREQQSRAAEQWQNPVQGVSCKEESGCPGSMERQPEEGDRSSPQAREA
QPPLEEVGEPGAERPPGGAASETNPEPSSQEKPGFLCGDKVPLVCNPLYQYLLAQKKQEQP

>Pm_Scfn2
MAHLLDKMCTIMGVFYKHAKQRGNCSALTRREMKTLLQEEFAGVIENPRDPETLELTFQLLDVNKDCLVDFNEYLFLIFRVAKACDSHLQPRERRIQRGEARRAPHEDELRGE
GRDHRQLREAEREGGYVCERRDSDRPRLRQQEGATRREGRRDHFPQEPVARSDQRSRQARDQDLRDDCGRHRPSRETQEWEDDCEDRPRGSDEQGEVETCQQTHECES
RRDEAGRHQLRRREPSREEDHQRQSWERDYDQRDCLPRGGGDRQRHSQEATQRGEDERRRQPKVTECQMEEEEDLRRPRVSGQQVGEESHTRRCAAQGIRSAYGRSRP
SCEPQELEDGGREDYVCEVERRSRRRDQRVADECWDQRSDRKPVDCDDECHSFRRDGERRSLSCDPEREQRRSQRCDAEHTERSMSDCRADERRSQGQYRPQSRGAELR
EHDVRESERRRRQPVDQDDRTRPYPCEPTTRAEVEDQRWSRVLECREGQRTGLHPSECEPADGERRRPQSCEYEPREGDRRRSQRHAQTPEEDGWGREECHQDEAQEEE
NLRRRRPLSGDLEPRDYESCRPQRREREPRGDGQRRQQYYDSEPLDRDVERRMPEAPESAQREGRRGRQQCYDSETLESRRNRPQSHYSESWEDERRRPEEADFSDDERE
RPQARQSLSRPREEDRYYSSVGGRRECDRGRRQEYESEPRESERRPRQRCEPEDYEERGQRYCTPGAREQEGSRPQRSCDTDPIGVDGRRCQTREVDPREQQRRARCRDTE

QRDRERNQTLRRDGEARQGDQRRNWSHDTDRRDSEQQRRTQSCADDVGDVEPRRRGMDLGISEQQRRGPSQSSGAADADQRRVQAREEDLRESQRRRVVPSGLEHE
ASQQRRPHCCDSESRERENRRQDLHDPEERSRESRAGNRRVSKPRHTQSCAFSCN

>Pm_Scfn3
MASLLDSIHDISRVFYKHALCHGDDFTLSRREMRRVIQEEFPEIIKNPCDPQTLELVFQQLEHNKEGLVDFNGYLRLLSNMSKACLYHWEARGDVRRDLCKGELQRNETIPRQ
LRNVEWKGSDDYEIGDSDRMGPHPFDGSRRREERRDSFLRESLTQIDERSCYRRDLESESDSESSSSSSSSSSSSSSSSEYLEKEDVESRRQSDKQDLQRDEAGSRGFVKRDNL
ERCHPSRRSQLREGDEREEGSTQRGDDRRQCRPPECYMEDEEENLRRPRQRGPREGERRRTQPRREDDEEESKEDREMKTLQRYGGRRRFLILEALRDDVKRKEPLEKGLE
GRRSQQVYCADSRESERRRFQSPEPALCDKAQRNQRYYVHEHLENESDRRRCQACRPGVREDNQKKEQNPDLEGFKRGEERRRSLQREVPSQEAIQRRQPCYPETPERDA
ERRRSHRQHCESEAFEREAERRRSLRGKPGLCADSLKQHCEVGRFERGAERKRSVRREVASQEDINGRRERYECEPLEGNDERRGPARG

C

>Pm_EDKM
MSSLYKAFTNMIERNSKANHKKVAETEKFQKSEFKKLIHQELSPVKRTSSNKYKHMKNLPDSDVELMNDKEVVPCVY

>Pm_PGLYRP3
MMASQEIILLFLCALSQASGCFKLITPSKWGAKPANCSEPLKDLPPEYVIIIHTAQNPCKTRAECSREVRNAQDYHLGLKGWCDVAYNFLIGEDGLVYEGRGWRNVGSHTYGY
NDLSLGIAFIGTFVERSPNQAAWKALKCLLDFSVKVGYLSPDYLVIAHSDVSNLVSPGEPIRSEISKWPNYRHN

>Pm_S100-A10_1
MPSQLEHAMENIMFTFHKFAGDKNYLTKEDLRQLMEKEVPGYMENQKDPMAIDRIMKNLEECRDGKVNFEGYLSLIAGLTNGCNEYYVKKMKPTGVKKY

>Pm_S100-A10_2
MPSQLEHAMENIMFTFHKFAGDKNYLTKEDLRQLMEKEVPGYMENQKDPMAIDRIMKNLEECRDGKVNFEGYLSLIAGLTNGCNEYYVKKMKPTGVKKY

>Pm_S100-A11_1
MSSKYPVGPTETERCIESLLAVFQRYAGRDGNACTLSKREFLTFMNTELASFSKNQKDPGVLDRMMKKLDMNNDGQLDFGEFLNLIGGLAQACHSQVLASPTAGVPQNHE
TPQFFGPSEPRPMSFPNIPKSSTSVSHQNTSHIRHGKARDPLFIYYFFLNKTDGLNTKVSLFSVQSSRMEAFDCTLARGLSHVRPLPQNSELPPLFSNLKISHFSPLETSVQAGF
YSTVR

>Pm_S100-A11_2
MSSKYPVGPTETERCIESLLAVFQRYAGRDGNACTLSKREFLTFMNTELASFSKNQKDPGVLDRMMKKLDMNNDGQLDFGEFLNLIGGLAQACHSQVLASPTASVPQNHE
TPQFFGPSEPRPMSFPNSPKSSTSVSHQNTSHIRHGKARDPLFIYYFFLNKTDGLNTKVSLFSVQSSRMEAFDCTLARGLSHVRPLPQNSELPPLFSNLKISHFSPLETSVQAGF
YSTVR

>Pm_S100-A12
MSKSKTQMQVLADQLIDVFHKYAGQSDTLDKQEFKKMILEQFPDCVECPRKKQAKEQLFNELDTNKDNVVNFEEWTRLVGRFLHCSHEKFHQQHGGQQQQQQQPQQQ

Figure S1. Amino acid sequences of proteins encoded by EDC genes of the common wall lizard. **(A)** Amino acid sequences of proteins encoded by single-coding exon EDC (SEDC) genes. Only a selected subset of corneous beta proteins (CBPs), which are discussed in the main text, are included here. **(B)** Amino acid sequences of S100 fused-type proteins (SFTPs). **(C)** Amino acid sequences of proteins encoded by other EDC genes of the wall lizard. The following amino acid residues are highlighted because they are either important for protein cross-linking or highly abundant in EDC proteins: cysteine residues (C) are potential sites of disulfide bonds; lysine (K) and glutamine (Q) are potential sites of transglutamination; glycine (G), proline (P) and serine (S) are highly abundant. Only S100A proteins encoded by genes flanking *PGLYRP3* and *SCFN1* are shown here. Pm, *Podarcis muralis*.

A

```
EDPCCC3p   PCCCSKCSKCGCSPCCCPKCSKCGCSPCCCPKCSKCGCSPCCCSKSPCYQCKCSPCYSSKTPCSKCGCSPCCCPK
peptide 1           CGCSPCCCPK   CGCSPCCCPK                                 CGCSPCCCPK

EDPCCC3p   CSKCGCSPCCCPKSPCSTCGCSPCSCSKCSKCGCSPCCCPKSPCSTCGCSPCSCPKCSKCGCSPCCCPKSPCSNC
peptide 1     CGCSPCCCPK                    CGCSPCCCPK                    CGCSPCCCPK
peptide 2                                             SPCSTCGCSPCSCPK

EDPCCC3p   ESSPCCCYTHVKYSIYVPCCCPRCVCARSALNRSCCLPCYRGQCYYNYSPCCCYGHSCGCGIY
peptide 3                                   SCCLPCYR
```

B

```
EDPCCC4    MTYGCGSCCNSCGYRNFCGPCCAPCCNSGCCHSGCCHSGCCSCCSPCCCSPCCCSPCCCSPCCCSPCCCSPCCSP
peptide 4   TYGCGSCCNSCGYR

EDPCCC4    CCSPCCNPCCGGWGCGSCCHPCCGY
```

C

```
LOR2       MSQQRQSGCCGSNRSPRCGRRGSGSGSSCCGGSSGGGSSCGRRGSGSSGCGGSSVGIAQVSGGSSCGRRGSGSSCCGG
peptide 5                                            RGSGSSGCGGSSVGIAQVSGGSSCGR
peptide 6                                             GSGSSGCGGSSVGIAQVSGGSSCGR

LOR2       SSVGIAQVSGGYGGSCCSSGGGSGQGVVIVSGGQGSSCCGSSGGIQQIRGSGCCSGGSSGGAVIIPAGVGGQSSGCCV

LOR2       GGGYGSGMGQQKIPVIDSGFGSGGIGCGGGSGGGLGGQSIIVPVPGSGGSGGCYGGGQQVIGSGISSASGCCSGDAGV

LOR2       GGVKVIGGGSGRVSPVCGGGVSGGGVKVIGGSGRVSPVCGGGVSGGGVKVIGGGSGRVSPVYGGGASCGGVKVIGGGS
peptide 7                                                           VSPVYGGGASCGGVK

LOR2       GRVSPVYGGGASCGGVKVIGGGSGRVSPVYGGGASCGGVKVIGGGSGRVSPVYGGGVSGGGVKVIGGGSGGGGSICGG
peptide 7    VSPVYGGGASCGGVK        VSPVYGGGASCGGVK        VSPVYGGGASCGGVK
peptide 8                                                                VIGGGSGGGGSICGG

LOR2       GVSGGGVKVIGGGSGGGGSICGGGLSDGGVKVVGGGSGRISPVYGGGVSGGGVKVVGGGSGGGGSICGGGLSGGGVKV
peptide 8  GVSGGGVK
peptide 9          VIGGGSGGGGSICGGGLSDGGVK
peptide 10                                        ISPVYGGGVSGGGVK

LOR2       IGGGSGRVSPVCGGGSGGQTIVVSGGSSGGCCGGASSGTSVVVGGGGSSQVKVPLVVPPCLGQTKQVTSLPPSCK
```

Figure S2. Mass spectrometry-based proteomic analysis confirms expression of EDPCCC and loricrin 2 proteins in toes of the green anole lizard. Proteins were extracted from the toes of the green anole lizard (*Anolis carolinensis*) and subjected to mass spectrometry-based proteomic analysis. Peptides corresponding to **(A)** EDPCCC3, of which only a partial sequence (EDPCCC3p) could be predicted from the genomic DNA sequence, **(B)** EDPCCC4 and **(C)** loricrin 2 (LOR2) were detected. The amino acid sequences of the proteins and peptides were aligned. In the protein sequences, arginine (R) and lysine (K) residues, corresponding to predicted cleavage sites of trypsin/LysC, are highlighted by red fonts. Sequences matching the peptides are marked by thin or thick underlines.

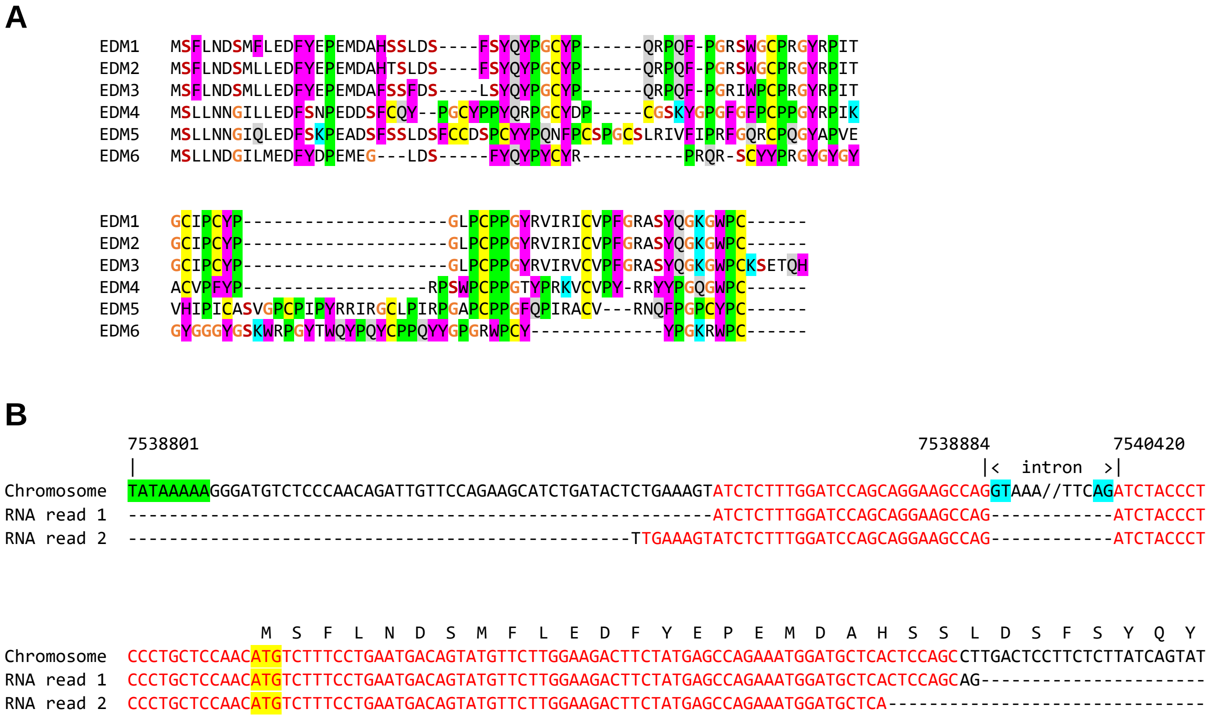

Figure S3. Amino acid sequence alignment of EDM proteins and evidence for expression of EDM1 in the skin of the common wall lizard. (A) Alignment of the amino acid sequences of EDM proteins of the common wall lizard (*Podarcis muralis*). Amino acid residues are colored as in Figure S1. In addition, the aromatic acids phenylalanine (F), histidine (H), tryptophan (W) and tyrosine (Y) are highlighted in magenta. Sequences were aligned with the MultAlin program (Corpet 1988) followed by manual adjustments. **(B)** RNA-seq evidence for expression of *EDM1*. Intron-spanning RNA-seq reads of a *P. muralis* skin transcriptome (Sequence read archive accession: SRX5274937; RNA read 1: SRR8468522.51120804, RNA read 2: SRR8468522.51120805) were aligned to the nucleotide sequence of the chromosomal locus of the *EDM1* gene (Chromosome). Sequence matches are indicated by red fonts. The first and last five nucleotides of the intron are shown, separated by //. The TATA box (green), splice site consensus sequences (blue) and the start codon (yellow) are highlighted. Numbers above the sequences indicate nucleotide positions (GenBank accession number NC_041327).
